# NetMHCpan-4.2: improved prediction of CD8+ epitopes by use of transfer learning and structural features

**DOI:** 10.3389/fimmu.2025.1616113

**Published:** 2025-08-07

**Authors:** Jonas Birkelund Nilsson, Jason Greenbaum, Bjoern Peters, Morten Nielsen

**Affiliations:** ^1^ Department of Health Technology, Technical University of Denmark, Lyngby, Denmark; ^2^ Center for Vaccine Innovation, La Jolla Institute for Immunology, La Jolla, CA, United States; ^3^ Department of Medicine, Division of Infectious Diseases and Global Public Health, University of California, San Diego, La Jolla, CA, United States

**Keywords:** HLA class I, MHC class I, antigen presentation, immunogenicity prediction, machine learning, immunoinformatics

## Abstract

**Introduction:**

Identification of CD8+ T cell epitopes is crucial for advancing vaccine development and immunotherapy strategies. Traditional methods for predicting T cell epitopes primarily focus on MHC presentation, leveraging immunopeptidome data. Recent advancements however suggest significant performance improvements through transfer learning and refinement using epitope data.

**Methods:**

To further investigate this, we here develop an enhanced MHC class I (MHC-I) antigen presentation predictor by integrating newly curated binding affinity and eluted ligand datasets, expanding MHC allele coverage, and incorporating novel input features related to the structural constraints of the MHC-I peptide-binding cleft. We next apply transfer learning using experimentally validated pathogen- and cancer-derived epitopes from public databases to refine our prediction method, ensuring comprehensive data partitioning to prevent performance overestimation.

**Results:**

Integration of structural features results in improved predictive power and enhanced identification of peptide residues likely to interact with the MHC. However, our findings indicate that fine-tuning on epitope data only yields a minor accuracy boost. Moreover, the transferability between cancer and pathogen-derived epitopes is limited, suggesting distinct properties between these data types.

**Discussion:**

In conclusion, while transfer learning can enhance T cell epitope prediction, the performance gains are modest and data type specific. Our final NetMHCpan-4.2 model is publicly accessible at https://services.healthtech.dtu.dk/services/NetMHCpan-4.2, providing a valuable resource for immunological research and therapeutic development.

## Introduction

Major Histocompatibility Complex class I (MHC-I) molecules play a pivotal role in the adaptive immune system. Anchored to the cell surface, MHC-I molecules bind and present peptide fragments derived primarily from degraded intracellular proteins. CD8+ T cells can interact with the peptide-MHC-I complexes, potentially initiating an immune response intended to kill off infected cells. Understanding the rules for MHC-I antigen presentation is thus crucial for rational development of disease treatments targeting CD8+ T cell activation.

Structurally, MHC-I molecules consist of an alpha chain and a beta-2-microglobulin chain. The alpha chain, encoded in humans by the HLA (Human Leukocyte Antigen) gene, is highly polymorphic and forms a peptide binding cleft that is closed at both ends. Due to the closed structure of the MHC-I binding cleft, a limited range of peptide lengths can be accommodated. While 8–11 mer peptides are most common, longer peptides can also bind by generally adopting a ‘bulging’ conformation in which certain residues protrude away from the binding cleft (1). Although these bulging residues may not interact with the MHC molecule, they are important for the peptide-MHC’s interaction with T cells.

In order to characterize the rules of MHC binding, binding affinity (BA) assays have historically been employed, in which each peptide’s affinity towards a single MHC-I molecule is measured *in vitro*. However, datasets produced by such assays by design do not contain information regarding the steps in the MHC class I presentation pathway leading up to the peptide presentation. The use of liquid chromatography coupled with mass spectrometry (LC-MS/MS) has resolved this issue by allowing for high-throughput generation of datasets describing the immunopeptidome. In these assays, peptide-MHC complexes are purified from cell lines in which one or more known MHC allotypes are present, after which the peptide ligands are retrieved and sequenced. These so-called eluted ligand (EL) datasets ultimately consist of lists of peptides known to bind to at least one of the MHC molecules expressed in the original cell lines. Cell lines are sometimes engineered to express only one MHC allele, in which case the immunopeptidome datasets are single-allelic (SA). However, for multi-allelic (MA) samples, it is necessary to assign each peptide to its most likely MHC target, a process commonly referred to as motif deconvolution.

To address this issue, Alvarez et al. have proposed the NNAlign_MA machine learning framework, which simultaneously deconvolutes immunopeptidome datasets and trains a pan-specific prediction model for MHC antigen presentation, allowing for highly improved motif deconvolution power (2). This framework has been incorporated into the widely used NetMHCpan-4.1 prediction method (3).

NNAlign_MA accommodates peptides of variable length by use of insertions and deletions. While insertions are used for peptides shorter than the motif length of 9, for peptides longer than 9, a 9-mer binding core is extracted either as a sub-sequence or by deleting a continuous stretch of amino acids reflecting potential bulging residues within the peptide. Until now, no information other than the length and position of the deletion has been used as an input feature to the NNAlign method. Furthermore, even though the deletions should ideally correspond to the residues bulging outwards in the peptide-MHC complex, no constraints have been placed on the position of the deletion so far. Recently, features related to MHC-peptide structures have been explored in publications about MHC-peptide binding (4) and peptide immunogenicity (5) prediction with great utility. Given this, we expect that integrating such structural features into NNAlign could therefore enhance its ability to more accurately model the interaction propensity of individual peptide residues and boost the overall predictive performance.

Beyond prediction of MHC antigen presentation, several tools have been developed for the direct prediction of immunogenicity (6–9). Training these tools directly from epitope data has proven challenging given the limited data available with accurate immunogenicity annotations of individual peptides (10). Given this, prediction of MHC-I antigen presentation is often used as a proxy for CD8+ epitope prediction. However, recently several publications have used the concept of transfer learning to refine methods trained on MHC ligands to predict epitopes more accurately, potentially resolving the issue of limited data volume, resulting in many cases in large proposed performance gains (11–13).

Given this background, we here seek to improve upon the previously developed NetMHCpan-4.1 method for prediction of both naturally presented ligands and epitopes. We achieve this by first integrating newly curated BA and EL data covering additional MHC-I alleles. Second, we investigate the use of an updated version of NNAlign_MA that incorporates new input features related to amino acid deletions. Finally, the obtained method is further refined using transfer learning on epitope data from the public domain in order to learn features specific to MHC-I epitopes and hence boost the predictive performance for their identification.

## Results

We set out to capitalize on the large amount of available MHC class I (MHC-I) peptide binding, immunopeptidome and epitope data in order to improve upon current state-of-the-art predictors, in particular for epitope prediction. To achieve this, we compiled a dataset of binding affinity (BA), MS eluted ligand (EL) and epitope data available in the public domain. We used the Immune Epitope Database (IEDB) to collect BA data points and identified a set of published MS immunopeptidomics datasets that were not used in the training of NetMHCpan-4.1, allowing us to expand both the BA and EL data. Furthermore, we collected positive and negative class I epitopes deposited in the IEDB (14), as well as a set of positive and negative neoepitopes from the CEDAR database (15). Then, variants of the NNAlign_MA machine learning framework used in NetMHCpan-4.1 were used to train a series of prediction models for predicting MHC class I antigen presentation and CD8+ epitopes.

### Integration of new EL training data

**Figure 1:**
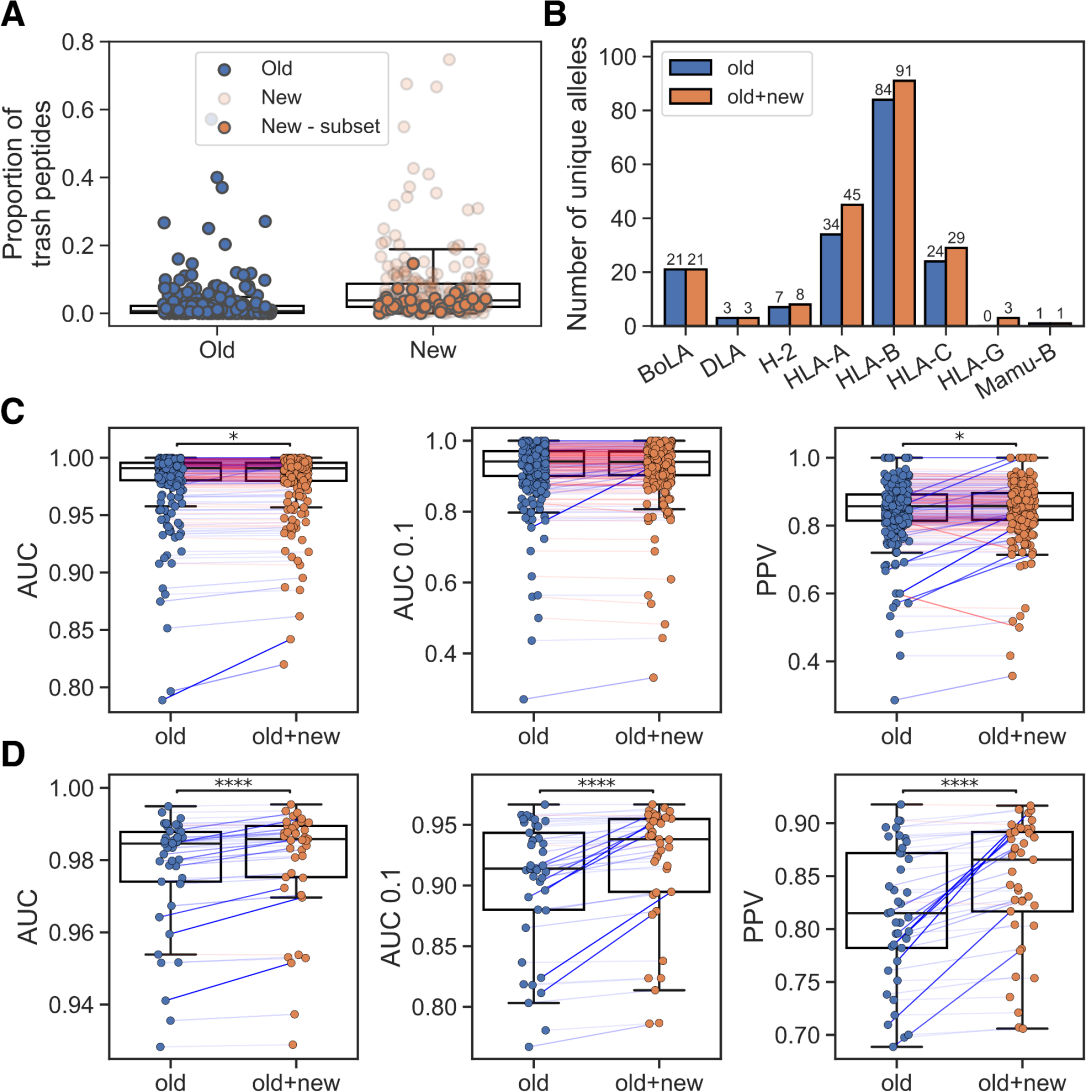
Integration of new EL data. **(A)** Percentage of trash peptides in old and new EL datasets. ‘New - subset’ refers to the chosen subset of EL datasets that covers alleles with low peptide count in the old EL training data. **(B)** Number of unique alleles per MHC-I locus represented in the training data without (old) and with the new EL data subset (old+new). **(C)** Performance on old EL samples for models trained without (old) and with the new EL data subset (old+new). **(D)** Performance on the new EL data subset for models trained without (old) and with this subset (old+new). In **(C, D)** pairings corresponding to greater than or equal performance in the old+new method are colored blue and otherwise red (with the line weight indicating the magnitude of the difference), and significant results from paired t-tests are shown (*p< 0.05, ****p< 0.0001).

As a baseline, we trained a method with the re-curated BA dataset and the EL data subset that was included in the training of NetMHCpan-4.1 (old EL). The model was trained in a cross-validation setup and evaluated in terms of AUC, AUC 0.1 and PPV (for details on these metrics and the model training see Materials and Methods). We then investigated the cross-validation predictions on the entire EL data using this model, including the new EL data not used during training. The performance on the new EL data was generally lower than the performance on the old EL data (median PPV 0.857 and 0.791, respectively). A major source of this reduced performance was a significant proportion of ‘trash’ peptides with %-rank greater than 20 in many of the new samples (see **Figure 1A**) compared to the old EL datasets. In **Supplementary Figure S1**, we show motif deconvolutions for several of these noisy datasets, illustrating that the predicted ‘trash’ peptides have length distributions and motifs that do not resemble those of predicted binders. Furthermore, when looking at the alleles covered by the new EL datasets, only a small proportion were either completely absent or had poor peptide coverage (<100 peptides with %-rank< 20) in the old EL data (see **Supplementary Table S1**).

Given this analysis, we decided to only include new EL datasets if they included at least one allele with a peptide count less than 100 in the old EL data, thus avoiding adding potentially low-quality datasets for alleles already covered in NetMHCpan-4.1. This yielded a subset of 41 datasets, of which 26 are SA and 15 are MA. An overview of the number of unique alleles represented in the old and combined EL data is shown in **Figure 1B**. We then trained a new model, again using cross-validation, including the new datasets that enriched the covered allelic space. While maintaining the overall performance on the old EL data (**Figure 1C**), a significant performance gain on the new included EL datasets was observed, as expected, for the model trained with these data (see **Figure 1D**, p<1.3*10–^5^ in all metrics, paired t-tests).

Comparing the MHCs covered by at least 100 peptides with %-rank less than 20, the method trained including the new EL data covered a total of 163 MHC molecules, compared to 130 in the method trained without these new data. Using the two sets of covered MHC molecules, we compared their estimated population coverage using data from the allelefrequencies.net database (16). Here, the extended set of 163 molecules was estimated to have a total coverage of ~96%, ~93% and ~97.3% for HLA-A, HLA-B and HLA-C, respectively, corresponding to percentage point increases of ~6.8%, ~11.4% and ~3.6%, respectively, when comparing to the model trained without the new data. This suggests that the new EL data yields a sizable increase in allelic coverage for all three loci. Interestingly, several non-covered HLA-B molecules, such as HLA-B*40:05 and HLA-B*35:43, were reported to have high allelic frequencies in large population sample sizes, and additional immunopeptidomics data covering these HLA-B alleles would therefore help to close the coverage gap between HLA-B and the other two HLA-I loci.

### Encoding of deletion composition and MHC interaction frequency

An important feature of the NNAlign_MA (and NNAlign) method is the inclusion of insertions/deletions to yield a common binding core length for all input peptides, regardless of length. For peptides of length > 9, such deletions ideally should correspond to residues bulging outwards in the peptide-MHC complex facing T cell interaction. This means that deleted residues potentially could share amino acid composition and/or structural properties enabling such protrusions, allowing for potential epitope T cell engagement. To investigate this, we modified the NNAlign_MA algorithm to encode novel features related to amino acid deletions. In short, peptide-MHC residue contacts were extracted from a set of peptide-MHC structures from the PDB database (17), and the frequency of MHC interactions for each peptide position was calculated as an average across all structures for the given peptide length. The average of these interaction values across the deleted positions, along with the average BLOSUM50 encoding of the deleted amino acids, was then encoded as additional inputs (for more details see Methods and Materials). The rationale was that by informing the model of the deleted residues’ amino acid composition along with how often the given residues’ positions on average interact with the MHC molecule, the method can make a more informed decision about where to place the deletion within the peptide.

**Figure 2:**
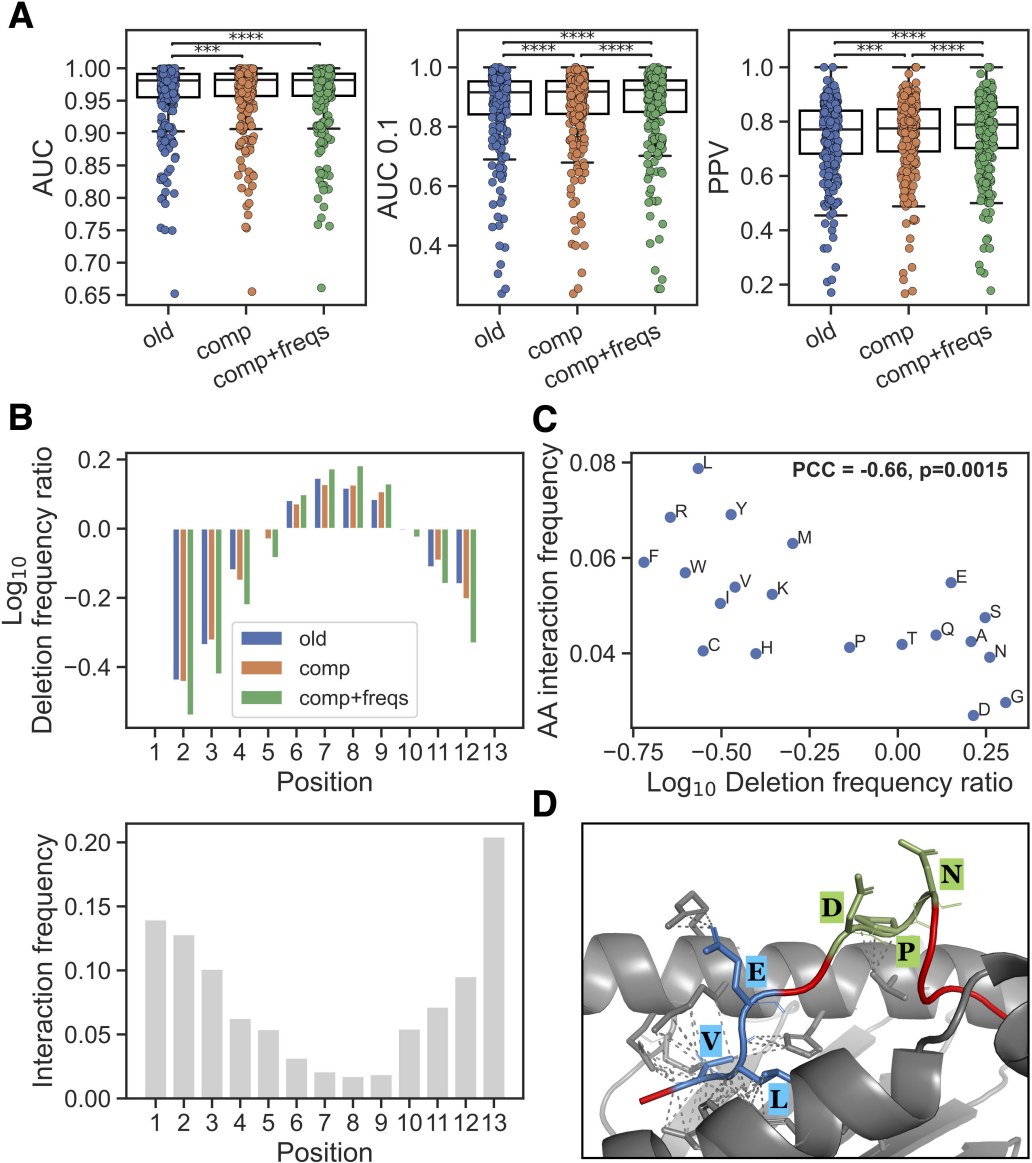
New input features and their impact. **(A)** Performance on peptides of length 10–14 in the EL training data for methods trained without new features (old), with average deletion composition (comp) and with average deletion composition and positional interaction frequency (comp+freqs). Each dot is an EL dataset, Significant results from paired t-tests are shown (***p< 0.001, ****p< 0.0001). **(B)** Top: Log_10_ ratio of observed frequencies for deletion placements in 13-mers divided by frequencies obtained through random sampling of deletions in the same peptide set, for the methods without new features (old), with average deletion composition (comp) and with average positional interaction frequency for deletion (comp+freqs). Bottom: the positional interaction frequency vector for peptide length 13 (which was used for the training of the comp+freqs method) is shown. **(C)** Relationship between observed peptide-MHC amino acid interaction frequencies and amino acid deletion. The x-axis is the log_10_ ratio between amino acid frequency in the comp+freqs and old model deletions in all positive 10–14 mers in the EL training data with a %-rank less than 20 evaluated in cross-validation. The y-axis is the normalized frequency of observed interaction between the given amino acid and an MHC molecule (for details see materials and methods). **(D)** The 12-mer peptide LVLEVDPNIQAV (red) and HLA-A*02:01 (grey) complex was modelled using ColabFold (19) v1.5.5 and visualized in PyMOL (20) (for more details see materials and methods). Deletions predicted by the old and comp+freqs models are highlighted and labeled in blue and green, respectively. Contacts between deleted residues and MHC residues within 4 Ångstrøm are shown with dashed lines.

Using this updated NNAlign_MA method, different models were trained and evaluated using cross-validation on the extended EL data set. Evaluating the impact of including only the deletion BLOSUM50 composition (*comp*), a small but consistent performance gain was observed for peptides of length 10-14 (p<0.001 in all metrics, paired t-tests). Furthermore, adding the positional interaction frequency input feature (*comp+freqs*) gave an additional gain in performance for these longer peptides (see **Figure 2A**, p< 4.9·10–^5^ for AUC 0.1 and PPV, paired t-tests). We further show the performance on a per peptide length basis in **Supplementary Figures S2**–**S8**, illustrating that while similar performance, as expected, was observed between all three methods for length 8 and 9, an improved performance was observed in the methods with the novel features across 10–14 mers.

We then checked if there was a difference in the placement of deletions for peptides of length > 9 after training with the new input features. Here, the cross-validated predictions for all positive 10–14 mers in the EL training data with a %-rank less than 20 were included [this to exclude potential MHC irrelevant co-immunoprecipitated contaminants from the analysis (18)], and the positional deletion frequencies were next compared to those obtained from a random sampling of deletions in the same peptide sets (see **Figure 2B**, **Supplementary Figure S9**). These results show that the *comp+freqs* method had a clear enrichment of deletions placed in the middle of the peptide. This corresponds well with the observed positional interaction frequency vectors which show that the middle positions are rarely interacting with the MHC (**Figure 2B** lower panel). It should be noted that, regardless of the input features used, deletions are found to be depleted near the peptide termini. This is expected due to the presence of MHC-I anchor positions in these areas.

Given that the new interaction frequency input feature is guiding the model to place its deletions at positions having generally less contact with the MHC, we hypothesized that the methods would learn to delete a different composition of amino acids. To investigate this, we did a simple analysis and calculated the log_10_ frequency ratio of each amino acid across all deleted residues in the peptide subset described above, comparing the model with the new features to the model without them. We then compared these log_10_ ratios to the frequency of MHC interaction for each amino acid in structures for peptides of length 10-14 (**Figure 2C**). For details on how these amino acid frequencies of MHC interaction were calculated, refer to materials and methods. Interestingly, we observed that the log_10_ deletion frequency ratios had a significant negative Pearson’s correlation with the MHC interaction frequencies (correlation=-0.66, p=0.0015, exact distribution test), indicating that residues which are more enriched in deletions by the method with the new features are observed to interact less with the MHC.

To complement this analysis, we investigated the source of the improved predictions in the method with the new features. Briefly, we looked at positive 10–14 mer peptides which had a lower %-rank score in the model with the new features compared to the model without these features, and where the %-rank score in the model with the new features was less than 2 (in order to focus on high-confidence binders). For each MHC molecule, we then constructed log_10_ deletion frequency ratios per amino acid and per peptide position and visualized these as heatmaps in **Supplementary Figures S10**–**S15**. The heatmaps reveal subtle differences between the different MHC molecules in their deletion enrichment patterns. We highlight these results, along with logo plots of the predicted binding cores for the methods without and with the new features, for HLA-A*02:01 in **Supplementary Figure S16**. Here, the model with the new features had an enriched deletion of amino acids such as glycine (G), which was reflected in the logo plots mainly at positions 4 and 5. Further, when looking at the per-position deletion frequencies in 10-mers, the highest enrichment of deletions is observed at P4.

As a final validation of the new features’ positive impact on the predictions, we selected one of the improved peptides from above binding HLA-A*02:01 (LVLEVDPNIQAV) which had different deletions predicted by the methods with and without the new features, and modeled the peptide-HLA complex using ColabFold (19) v1.5.5. Note, that this peptide was not part of any of the PDB structures used to define the interaction frequency input feature. As illustrated in **Figure 2D**, the method with the new features predicted a deletion of positions 6-8 (DPN, shown in green) which are bulging outwards from the MHC binding cleft, with two out of three residues not having any contact with the MHC using a distance threshold of 4 Ångstrøm. On the other hand, the method without the new features predicted a deletion of positions 2-4 (VLE, shown in blue) which are all in contact with the MHC, again using a threshold of 4 Ångstrøm. Together, these results suggest that the model with the new features has learned biologically relevant information and can more accurately identify which amino acids are relevant for the peptide’s interaction with the MHC and which are bulging outwards from the binding cleft facing interaction with potential T cell receptors.

### Incorporation of peptide context

For MHC class II, residues flanking the peptide in the source protein have been shown to contain important information regarding antigen processing, and incorporating this peptide context information into the NetMHCIIpan methods has been shown to boost antigen presentation prediction performance (21). To investigate to what degree a similar approach could boost the performance for class I, the comp+freqs model described above was retrained using peptide context and evaluated in the same cross-validation setup. As described earlier for MHC class II (21), peptide context was here defined as a 12-mer sequence consisting of three residues flanking the peptide’s N- and C-terminal in the source protein, along with the first three residues in the peptide’s N- and C-terminal. In line with previous results for MHC class II, a highly significant performance increase was observed for the model including context across all metrics (p<2.4·10–^12^ in all cases, paired t-tests, see **Supplementary Figure S17**). In line with previous research (22, 23), however, inclusion of peptide context did not yield improved performance for prediction of CD8+ epitopes (see **Supplementary Figure S18**).

### Model refinement on epitope data

Having arrived at an updated model that improves performance on prediction of antigen presentation, we next focused on the uttermost important task in the context of MHC class I, namely epitope prediction. Specifically, we sought to improve the model’s ability to identify epitopes through transfer learning by refining the model trained to predict antigen presentation on experimentally validated epitopes. We employed a set of epitope and neoepitope data downloaded from the IEDB and CEDAR databases, respectively. The datasets were initially merged with the EL and BA training data and divided into cross-validation partitions, ensuring no peptide overlap between partitions. Then, epitope subsets of these datasets were extracted for training and testing, such that there was no 8-mer overlap between the test and training epitopes. Using these datasets, we investigated how refining on each dataset (IEDB vs CEDAR) impacted the model’s ability to predict the unseen test set epitopes from either database. As baselines, we used predictions from NetMHCpan-4.1, the non-refined version of the *comp+freqs* method, as well as models trained on the epitope training sets with NNAlign-2.1 (24). For more details on the training and test set construction and the NNAlign baselines, refer to materials and methods.

**Figure 3:**
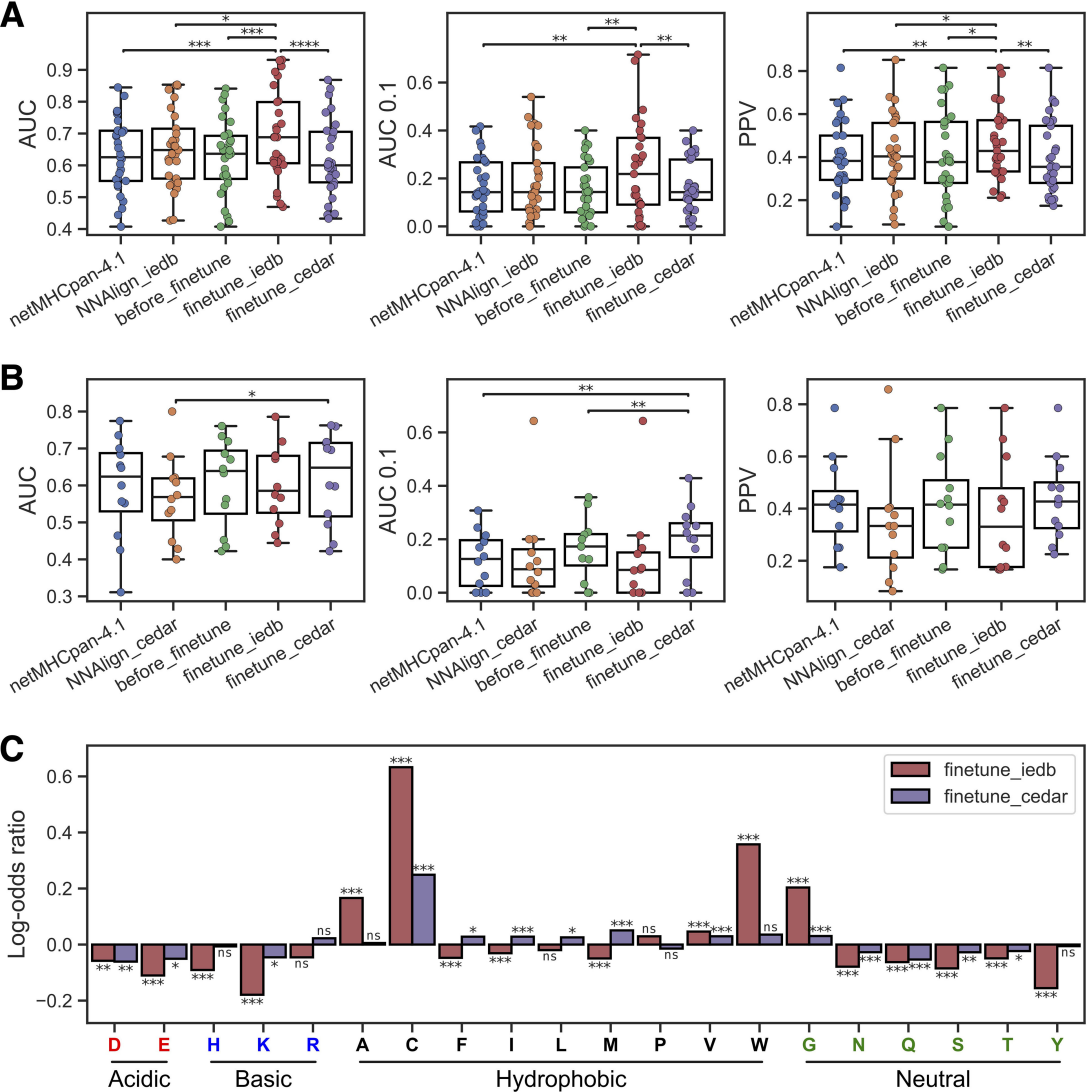
Transfer learning yields improved epitope and neoepitope prediction. **(A)** Performance on the IEDB test set for netMHCpan-4.1, NNAlign-2.1 trained on IEDB (NNAlign_iedb), our non-refined method (before_finetune), and our methods refined on IEDB (finetune_iedb) and CEDAR (finetune_cedar). **(B)** Performance on the CEDAR test set for netMHCpan-4.1, NNAlign-2.1 trained on CEDAR (NNAlign_cedar), our non-refined method (before_finetune), and our methods refined on IEDB (finetune_iedb) and CEDAR (finetune_cedar). In **(B, C)** each dot is an MHC-I molecule, and significant results from paired t-tests are shown (*p< 0.05, **p< 0.01, ***p< 0.001, ****p< 0.0001). **(C)** Log-odds ratios of average amino acid frequencies in the top 1% of 125,000 random 8–12 mer peptides across all MHC molecules with unique pseudo-sequences in the IEDB and CEDAR training data. Each bar is equal to the log_10_ of the average frequency in the refined method divided by the average frequency in the non-refined method. The result from a permutation test with 10,000 replications between the amino acid frequency vectors across MHCs is shown above each bar (ns, not significant, *p< 0.05, **p< 0.01, ***p< 0.001, p-values are adjusted for multiple testing using Benjamini-Hochberg correction). Amino acids are grouped by the properties of their side chains.

Evaluating first the performance on the IEDB test set, we observed that the model fine-tuned on IEDB epitopes had the highest overall performance, with a significant gain over all the baseline methods (**Figure 3A**). However, this performance gain was not present for the model refined on the CEDAR data. Here, a performance loss was observed compared to the non-fine-tuned model. A similar observation was found for the models evaluated on the CEDAR test data. Here, a significant gain in AUC compared to the NNAlign baseline, as well as a significant gain in AUC 0.1 over NetMHCpan-4.1 and our new non-refined method, was observed for the method refined on the CEDAR neoepitopes (**Figure 3B**). However, the model fine-tuned on IEDB data demonstrated a performance loss compared to the baseline models for this evaluation data set. These observations indicate that the transfer learning approach works favorably only when applied to the test set from the same data source used for the fine-tuning, suggesting that the features defining immunogenicity in the IEDB and CEDAR datasets differ and do not transfer to each other.

To further investigate this observation and understand what the refined models had learned from the epitope and neoepitopes, we looked at the differences in amino acid composition of high-ranking peptides in each method. Here, the amino acid composition of the top 1% of 125,000 random 8–12 mer peptides across all MHC molecules with unique pseudo-sequences (25) in the IEDB and CEDAR training data MHCs was calculated for both the refined methods and the non-refined method, and the log-odds ratio between the amino acid frequencies in the refined methods and the non-refined methods were visualized in **Figure 3C**.

Comparing these amino acid compositions, we observed a significantly higher proportion of tryptophan (W) in the IEDB-refined predictions compared to the non-refined method (Benjamin-Hochberg adjusted p=0.0004, permutation test with 10,000 resamples). Likewise, we find that this enrichment is absent from the CEDAR refined method, aligning with findings in previous research on epitope immunogenicity (7). Furthermore, both the IEDB and CEDAR refined methods had an increased frequency of cysteine (C) than the non-refined method (Benjamin-Hochberg adjusted p=0.0004 in both cases, permutation tests with 10,000 replications). Cysteine is underrepresented in MS-based immunopeptidomics data (26), but not so in epitopes, and therefore fine-tuning on epitopes has at least in part corrected for this bias.

A similar analysis as above was done in order to investigate potential compositional differences in amino acid deletions across the different methods (see **Supplementary Figure S19**). Here, we observed that hydrophobic amino acids were highly enriched among deletions in both the IEDB-refined and CEDAR-refined methods compared to that of the non-refined method.

Overall, refinement on neoepitopes yielded a smaller performance gain on the CEDAR test set in comparison to the IEDB test performance after IEDB epitope refinement. This is likely at least in part due to the smaller neoepitope training dataset size, with only 3,297 CEDAR data points compared to 23,891 IEDB data points. As a side remark, inclusion of peptide context in the context of model refinement also did not yield improved performance after refinement for both the IEDB and CEDAR datasets (see **Supplementary Figure S18**).

Overall, these findings suggest that the refined methods have captured signals defining the different amino acid compositions between MS identified antigen presented peptides and epitopes, and that these differences serve as a driving factor behind the improved predictive performance after fine-tuning on data from IEDB and CEDAR.

### NetMHCpan-4.2

The final models were implemented as a webserver available at https://services.healthtech.dtu.dk/services/NetMHCpan-4.2. Predictions can be made in three ‘modes’, namely antigen presentation prediction (EL and BA method), pathogen epitope prediction (IEDB fine-tuned method), and neoepitope prediction (CEDAR fine-tuned method). Furthermore, an option to include peptide context is available for the antigen presentation method.

## Discussion

Here we aimed to enhance the predictive accuracy of MHC-I antigen presentation and epitope identification by integrating newly curated binding affinity (BA) and eluted ligand (EL) datasets, refining the NNAlign_MA machine learning framework, and leveraging transfer learning for epitope prediction.

The inclusion of newly curated EL data sets yielded an increase in both the number of unique MHC alleles covered and their estimated population coverage based on allelic frequency data, improving our method’s worldwide applicability. Furthermore, the use of amino acid deletion composition and interaction frequency as input features to the NNAlign_MA method significantly enhanced prediction accuracy for longer 10–14 mer peptides. When investigating the deletions by our method in these peptides, the amino acid composition was found to be strongly inversely correlated with the composition of residue interactions in peptide-MHC protein structures from the PDB. Importantly, this correlation was found to be absent for the model trained without this additional input, suggesting that the new model has captured essential structural properties associated with the positional and amino acid compositional importance of residues interacting with the MHC and residues facing out towards the T cell receptor.

Our study also explored the incorporation of peptide context in MHC-I binding predictions, a feature previously demonstrated to be important for MHC-II predictions. The observed improvement in predictive performance across all evaluation metrics suggests that flanking residues contribute meaningfully to MHC-I antigen processing and presentation. However, and in line with earlier work, this performance gain did not carry over to the prediction of CD8 epitopes.

Refinement of the predictive model using transfer learning on epitope datasets of pathogen (from the IEDB) or cancer (neo-epitopes from CEDAR) origin demonstrated that epitope-specific features could be learned to enhance immunogenicity predictions. The performance gain observed in IEDB and CEDAR test sets after dataset-specific fine-tuning however demonstrated a dataset-specific nature of epitope prediction. That is, methods refined on pathogen derived epitopes did not demonstrate improved performance on neo-epitopes and vice versa. Investigating the amino acid enrichment among predicted binders revealed subtle differences in amino acid preferences between the IEDB- and CEDAR-refined methods, suggesting that these differences may prevent performance improvement on opposite datasets. One clear example of this is the enrichment of tryptophan (W), which was only present in the IEDB-refined method, aligning well with previous studies which have shown a lack of tryptophan enrichment in neoepitopes (7). However, both refined methods shared a clear enrichment of cysteine, which likely stems from the underrepresentation of cysteine in MS immunopeptidomics data which is not present in the IEDB or CEDAR epitope datasets.

Overall, especially for neoepitopes, the performance gains after transfer learning were limited. This finding is in contrast to previous publications which have claimed large performance improvements over antigen presentation predictions methods through neo-epitope transfer learning (11, 12). Our results thus call into question potential issues of performance overestimation of these earlier studies, and the extent to which the current availability of validated epitope data is sufficient to significantly improve predictive accuracy of machine learning methods.

In conclusion, our findings illustrate the benefits of integrating high-quality immunopeptidome data, refining computational frameworks, and leveraging transfer learning to improve MHC-I antigen presentation and epitope prediction. The final model is publicly available at https://services.healthtech.dtu.dk/services/NetMHCpan-4.2, facilitating broader application in immunological research.

## Materials and methods

### Allelic frequencies

Allelic frequencies for HLA-A, HLA-B and HLA-C were obtained from the allelefrequencies.net database (16). Only datasets with the gold or silver standard were considered, which includes datasets with at least two-digit HLA typing. The allele frequencies were then calculated as an average over world-wide populations of size 100 and above, weighted by population size capped at a maximum value of 10,000. For a complete list of the calculated frequencies, see **Supplementary Table S2**.

### Binding affinity data

Binding affinity (BA) data was sourced from the Immune Epitope Database (IEDB) and includes 197,504 measurements covering 173 MHC-I molecules. Of these, 8,624 measurements were not included in the NetMHCpan-4.1 BA training data. The IC50 affinity values were transformed as described earlier (27) into the [0,1] range using the following relation:


$$f\left(x\right)=1-\frac{\log\left(x\right)}{\log\left(50,000\right)}$$

Furthermore, artificial negative BA data points with assigned target values of 0.01 were generated for each allele by sampling 100 random peptides of length 8–14 from the given allele’s organism’s proteome, ensuring that none of the sampled peptides were present in the true BA data.

### Eluted ligand data

As a starting point, we collected the eluted ligand (EL) training data from NetMHCpan-4.1, keeping all the positive peptides of length 8-14. We then queried the Immune Epitope Database (IEDB) for MHC class I immunopeptidomics datasets derived from recent publications not included in NetMHCpan-4.1 and with at least 1000 ligands listed in the IEDB. These immunopeptidome datasets were extracted manually from each publication, keeping only samples with full HLA typing and including all peptides of length 8–14 without post-translational modifications. The new EL datasets were combined with the NetMHCpan-4.1 training data for further data processing described below.

The peptide ligands from each EL dataset (both from the newer publications and the NetMHCpan-4.1 training data) were mapped to the proteome of the given source organism in order to define ligand context. Around 2.2% of the ligands could not be mapped and were thus discarded.

After removing 96,404 {peptide, HLA type} combinations in the new EL data which were also present in the NetMHCpan-4.1 EL data, each EL dataset was then supplemented with random negative peptides derived from the proteome of the given sample’s source organism. This enrichment was done in a per-sample id manner by uniformly sampling 8–14 mer peptides in an amount equal to five times the number of ligands for the most common peptide length in the given sample. By sampling the negatives from the same proteome as the positives for each dataset, we ensured that no information was leaked between samples of different source organisms. Furthermore, since the sampling was done uniformly across peptide lengths, we ensured that differences in peptide length distribution across datasets would not affect the learning.

Before filtering away new EL samples not used during training (see below), the EL dataset consists of 1,509,068 positive ligands and 31,595,388 random negative peptides across 465 samples. Of these, the new EL data consists of 861,258 positives and 18,276,213 negatives across 215 samples.

Only a subset of the new EL samples were ultimately included in the training and evaluation. These datasets had alleles with peptide counts less than 100 (excluding peptides with %rank > 20 in the cross-validation) in the old EL data. A total of 41 new EL samples from nine publications were included here (28–36). An overview of the entire training dataset can be found in **Supplementary Table S3**. Furthermore, an overview of the EL datasets not included in our final training data set is included in **Supplementary Table S4**. The entire EL training dataset consists of 792,905 positives and 16,556,256 negatives, of which 145,095 positives and 3,237,081 negatives are from the newly included EL samples. In total, the EL training data covers 201 MHC class I molecules, of which 27 are completely unique to the newly included EL samples.

### Epitope data

The IEDB was queried for positive and negative class I epitope assays. Only epitopes of length 8–14 with MHC allele typing, no post-translational modifications and with known source protein were considered. Each epitope-allele pair was labeled a positive if that pair had at least one positive assay, and otherwise negative. In order to investigate the impact of peptide context encoding on epitope prediction, only epitopes which could be mapped to their annotated source protein were included. This led to a total of 42,921 data points (10,302 positives and 32,619 negatives).

### Neoepitope data

A set of neoepitopes were downloaded from the Cancer Epitope Database and Analysis Resource (CEDAR), keeping only neoepitopes of length 8–14 with HLA allele typing. Similarly to the IEDB data, {neoepitope, HLA allele} pairs with at least one positive assay were labeled as positive, and the rest as negatives. Furthermore, the wild-type peptide variants of the neoepitopes were mapped to the human proteome to define peptide context. Here, ~1.7% of the wild-type peptides could not be mapped and the corresponding neoepitopes were therefore discarded. This resulted in a total of 5172 data points (1188 positives and 3984 negatives).

### Data partitioning

The EL, BA, epitope and neoepitope datasets were merged and split into five cross-validation partitions using the common motif approach (37), ensuring that peptides sharing at least an 8-mer overlap were placed in the same partition.

### Peptide-MHC interaction frequency

A set of peptide-MHC-I structures were downloaded from the Protein Data Bank (PDB) (17). For each structure, we required that its accompanying FASTA file contained a peptide of length 10–14 with standard amino acids. After filtering away structures with other protein chains than the MHC (e.g. T-cell receptors and antibodies), a total of 213 structures were retrieved (a complete list of the included structures is given in **Supplementary Table S5**). For each structure, we counted how many times each peptide position’s residue was within 4 Ångstrøm of a residue in the MHC molecule, ensuring that only positions with standard amino acids contributed. To counteract the low number of available structures for longer peptides, a pseudocount of 1 was added to each position in every count vector. Each count vector was then transformed to a frequency vector by dividing each element by the count vector sum. Finally, the average frequency vector for each peptide length across the included structures was calculated. For each peptide length, the average frequency vector thus indicates how often each peptide position is in contact with the MHC (regardless of which residue is in the given peptide position) across the structures with peptides of the given length.

A per-amino acid frequency of interaction was used to investigate the correspondence between peptide amino acid deletions and the deleted residues’ propensity to interact with the MHC molecule. This was done by first counting for each amino acid how many peptide-MHC residue contacts within 4 Ångstrøm were observed across all structures and normalizing that count by the number of PDB structures that included these contacts. These normalized counts were then transformed to frequencies by dividing by the sum across all amino acids.

### Training on EL and BA data

All models were trained with the NNAlign_MA machine learning framework (2) using either the original method or a modified version (see below). We used a similar training setup to that used for NetMHCpan-4.1 (3). That is, each model consists of an ensemble of 100 neural networks each with one hidden layer holding either 56 or 66 hidden neurons, with 10 random weight initializations for each of the 5 cross-validation folds (2 architectures, 10 seeds, and 5 folds). The weights were initialized randomly as either -0.1 or 0.1. All models were trained using backpropagation with stochastic gradient descent using a constant learning rate of 0.05, for 200 epochs with early stopping. Only single allele (SA) data were included in the training for a burn-in period of 20 epochs. Subsequent training cycles included multi-allele (MA) data.

To investigate the performance impact of the new EL data, two initial models were trained with the original NNAlign_MA method. One of these models used BA data along with only the EL data found in the NetMHCpan-4.1 training data. The other model included new EL datasets that contained alleles covered with less than 100 peptide annotations (with %-rank< 20) in the older NetMHCpan-4.1 EL data.

The NNAlign_MA machine learning method was next modified to include new encoding options related to amino acids deletions. Firstly, the deletion composition was encoded with 20 input values corresponding to the average BLOSUM50 encoding vector of the deleted residues. Furthermore, the average positional MHC interaction frequency from the vectors described earlier was encoded with two input values, *average frequency* and *1 - average frequency*. For 8- and 9-mer peptides, no deletions are performed, and in these cases the novel features were instead represented by 20 zeros for the average BLOSUM50 deletion encoding, and [0,1] for the average positional interaction frequency encoding. The same was done for 10+ mers for which a 9-mer sub-sequence without deletions is selected as the binding core.

Another set of models were trained to investigate the impact of the new input features. The first of these included the deletion composition, and the second one included both the deletion composition and the average positional interaction frequency. The model with both new input features was included in the NetMHCpan-4.2 method as the base EL/BA prediction method.

The final model described above was also trained with peptide context encoding. Here, peptide context refers to three residues flanking the peptide’s N- and C-terminal in the source protein, along with the first three residues in the peptide’s N- and C-terminal, all concatenated into a sequence of 12 amino acids. In cases of missing context residues due to the peptide being located near the ends of the protein sequence, each missing residue was represented by X and encoded with 20 zeros. An overview of the final NetMHCpan-4.2 model architecture is shown in **Supplementary Figure S20**.

### Structural modelling and visualization

The 12-mer peptide LVLEVDPNIQAV in complex with HLA-A*02:01 was modelled using ColabFold (19) v1.5.5 (accessed at https://colab.research.google.com/github/sokrypton/ColabFold/blob/main/AlphaFold2.ipynb). The query sequence was set as the full-length HLA-A*02:01:01:01 sequence spanning positions 25 to 204 [as obtained from the IPD-IMGT/HLA database (38)] followed by a colon and the peptide sequence LVLEVDPNIQAV. All other parameters were set as default. Among the five models generated, the top-ranked model was selected and visualized using PyMOL (20) version 3.0.3.

### Fine-tuning on epitope data

The models obtained from training on EL and BA were used as a starting point to transfer learn epitope prediction. In order to fairly evaluate the impact of the fine-tuning, training and external test sets were made as described below.

For each of the IEDB and CEDAR datasets, we constructed an external test set with at least 5 positives and 5 negatives for each allele. This was done by first considering the set of epitopes without 8-mer overlap to the EL data, BA data and epitope data from the opposite source (such that none of the selected IEDB test epitopes would overlap with any potential CEDAR training epitopes and vice versa). Then, for each allele in this epitope set, we added 2/3 of the positives and negatives to the test set if this fraction corresponded to at least 5 positives and 5 negatives. This was done to ensure that alleles in the external test set were also represented in the training data. All other epitopes were then assigned to their respective cross-validation partition if they did not have an 8-mer overlap to the constructed test set. This resulted in test sets of 10,621 IEDB epitopes (2,298 positives and 8,323 negatives) and 1,486 CEDAR neoepitopes (315 positives and 1,171 negatives). The reduced training sets (each consisting of five cross-validation partitions) then consisted of 23,891 IEDB data points and 3,297 CEDAR data points.

The final models obtained from training on EL and BA data were fine-tuned by continuing the training on the BA and epitope/neoepitope training and validation partitions, where each model was trained for up to 100 epochs, applying early stopping. For the epitope and neoepitope training partitions, we applied label smoothing by assigning target values of 0.95 and 0.05 instead of 1 and 0 to positives and negatives, respectively, in order to reduce overfitting. Furthermore, for the CEDAR refinement we used a focal loss function for the neoepitope predictions with alpha=0.5 and gamma=1.0 (39), and applied a burn-in period of 20 epochs in which we upsampled positive neoepitopes to have an even proportion of positive and negative neoepitopes for training.

The performances of the refined models were compared with baseline models trained using NNAlign-2.1 (24). For both the IEDB and CEDAR datasets, an ensemble of 25 models was trained corresponding to five random seeds for each CV partition. Each network had 10 hidden neurons, and the same input features except for the new features introduced in this manuscript were used. The models were trained for 100 epochs with early stopping and with the burn-in option turned off.

The final refined models included in NetMHCpan-4.2 were fine-tuned on the entire IEDB or CEDAR epitope training sets without removed data points for the purpose of external test set construction, using the same training procedure as described above.

### Performance evaluation

Predictive performance was evaluated in a per-sample or per-allele manner in terms of the area under the Receiver Operating Characteristic (ROC) curve (AUC), area under the ROC curve integrated up to a false positive rate of 10% (AUC 0.1) and positive predictive value (PPV). Here, PPV is defined as the fraction of true positives in the top N predictions, where N is the number of positives for the given sample or allele. For the EL performance evaluation, only samples with at least 5 positive peptides were included. We are aware that using the same test fold for both early stopping and performance evaluation can in some cases lead to performance overestimation. However, we show in **Supplementary Figure S21** that training our final models without early stopping yields virtually the same cross-validated EL performance, thus justifying that the degree of performance overestimation is very limited if at all present.

### Data visualization

Data visualizations were created in Python 3.12.9 using the matplotlib (version 3.10.1) and seaborn (version 0.13.2) libraries. Sequence logos were generated with Seq2Logo-2.0 (40).

## Data Availability

Publicly available datasets were analyzed in this study. This data can be found here: https://services.healthtech.dtu.dk/services/NetMHCpan-4.2/ (under ‘Training data sets’).

## Supplementary material

Supplementary Figure 1 Motif deconvolution reveals high levels of ‘trash’ peptides in new datasets. Motif deconvolutions were performed for four newly analyzed single-allele datasets corresponding to HLA-A29:02, HLA-B54:01, HLA-B57:01, and HLA-C04:01, based on cross-validation predictions. The datasets were obtained from Sarkizova et al., 2020 (PMID: 31844290) and Mei et al., 2020 (PMID: 32357974). For each dataset, peptides with a %-rank >20 were annotated as ‘trash’, while those with a %-rank ≤20 were annotated as binders for the corresponding HLA molecule. Each row represents one dataset and shows, from left to right: the number of peptides assigned to the HLA and trash categories, peptide length distributions, the motif of predicted binders, and the motif of predicted trash peptides.

Supplementary Figure 2 Cross-validated performance on peptides of length 8 for methods trained without new features (old), with average deletion composition (comp) and with average positional interaction frequency for deletion (comp+freqs). Each point is a dataset from the EL training data. Significant results from paired t-tests are shown (*p< 0.05).

Supplementary Figure 3 Cross-validated performance on peptides of length 9 for methods trained without new features (old), with average deletion composition (comp) and with average positional interaction frequency for deletion (comp+freqs). Each point is a dataset from the EL training data. Significant results from paired t-tests are shown (*p< 0.05).

Supplementary Figure 4 Cross-validated performance on peptides of length 10 for methods trained without new features (old), with average deletion composition (comp) and with average positional interaction frequency for deletion (comp+freqs). Each point is a dataset from the EL training data. Significant results from paired t-tests are shown (*p< 0.05).

Supplementary Figure 5 Cross-validated performance on peptides of length 11 for methods trained without new features (old), with average deletion composition (comp) and with average positional interaction frequency for deletion (comp+freqs). Each point is a dataset from the EL training data. Significant results from paired t-tests are shown (*p< 0.05).

Supplementary Figure 6 Cross-validated performance on peptides of length 12 for methods trained without new features (old), with average deletion composition (comp) and with average positional interaction frequency for deletion (comp+freqs). Each point is a dataset from the EL training data. Significant results from paired t-tests are shown (*p< 0.05).

Supplementary Figure 7 Cross-validated performance on peptides of length 13 for methods trained without new features (old), with average deletion composition (comp) and with average positional interaction frequency for deletion (comp+freqs). Each point is a dataset from the EL training data. Significant results from paired t-tests are shown (*p< 0.05).

Supplementary Figure 8 Cross-validated performance on peptides of length 14 for methods trained without new features (old), with average deletion composition (comp) and with average positional interaction frequency for deletion (comp+freqs). Each point is a dataset from the EL training data. Significant results from paired t-tests are shown (*p< 0.05).

Supplementary Figure 9 Log_10_ ratio of observed frequencies for deletion placements in 10-14-mers divided by frequencies obtained through random sampling of deletions in the same peptide set, for the methods without new features (old), with average deletion composition (comp) and with average deletion composition and positional interaction frequency for deletion (comp+freqs). Below each log_10_ frequency ratio plot, the positional interaction frequency vector for the given peptide length (which was used for the training of the comp+freqs method) is shown.

Supplementary Figure 10 Log_10_ deletion frequency ratio per amino acid across alleles, calculated between the method with the new features and the method without the new features per amino acid. The ratios were calculated based on 10–14 mer peptides with improved %-rank in the method with the new features compared to the method without, and where the %-rank for the method with the new features was less than 2. Only alleles with at least 100 peptides were included.

Supplementary Figure 11 Log_10_ deletion frequency ratio per position in 10-mer peptides, calculated between the method with the new features and the method without the new features per amino acid. The ratios were calculated based on peptides with improved %-rank in the method with the new features compared to the method without, and where the %-rank for the method with the new features was less than 2. Only alleles with at least 100 peptides were included.

Supplementary Figure 12 Log_10_ deletion frequency ratio per position in 11-mer peptides, calculated between the method with the new features and the method without the new features per amino acid. The ratios were calculated based on peptides with improved %-rank in the method with the new features compared to the method without, and where the %-rank for the method with the new features was less than 2. Only alleles with at least 100 peptides were included.

Supplementary Figure 13 Log_10_ deletion frequency ratio per position in 12-mer peptides, calculated between the method with the new features and the method without the new features per amino acid. The ratios were calculated based on peptides with improved %-rank in the method with the new features compared to the method without, and where the %-rank for the method with the new features was less than 2. Only alleles with at least 100 peptides were included.

Supplementary Figure 14 Log_10_ deletion frequency ratio per position in 13-mer peptides, calculated between the method with the new features and the method without the new features per amino acid. The ratios were calculated based on peptides with improved %-rank in the method with the new features compared to the method without, and where the %-rank for the method with the new features was less than 2. Only alleles with at least 100 peptides were included.

Supplementary Figure 15 Log_10_ deletion frequency ratio per position in 14-mer peptides, calculated between the method with the new features and the method without the new features per amino acid. The ratios were calculated based on peptides with improved %-rank in the method with the new features compared to the method without, and where the %-rank for the method with the new features was less than 2. Only alleles with at least 100 peptides were included.

Supplementary Figure 16 Impact of new features on motif deconvolution for HLA-A02:01. The plots are based on peptides with improved %-rank in the method with the new features compared to the method without, and where the %-rank for the method with the new features was less than 2. **(A)** Sequence logos of predicted binding cores for the methods without and with the new features. **(B)** Log_10_ deletion frequency ratio per amino acid (left) and per position in 10-mer peptides (right), calculated between the method with the new features and the method without the new features.

Supplementary Figure 17 Cross-validated performance of models trained without and with context encoding. Each point is a dataset from the EL training data. Pairings corresponding to greater than or equal performance in the w_context method are colored blue and otherwise red (with the line weight indicating the magnitude of the difference). Significant results from paired t-tests are shown (****p< 0.0001).

Supplementary Figure 18 Performance of models without and with context encoding on external epitope and neoepitope test sets. **(A)** Performance on external IEDB test set before (wo_context_before, w_context_before) and after (wo_context_finetune_iedb, w_context_finetune_iedb) fine-tuning on the IEDB training data. **(B)** Performance on external CEDAR test set before (wo_context_before, w_context_before) and after (wo_context_finetune_cedar, w_context_finetune_cedar) fine-tuning on the CEDAR training data. In **(A, B)**, significant results from paired t-tests are shown (*p< 0.05, **p< 0.01, ***p< 0.001).

Supplementary Figure 19 Log-odds ratios of average amino acid frequencies in the deletions of top 1% of 75,000 random 10–12 mer peptides across all MHC molecules with unique pseudo-sequences in the IEDB and CEDAR training data. Each bar is equal to the log_10_ of the average frequency in the refined method divided by the average frequency in the non-refined method. The result from a permutation test with 10,000 replications between the amino acid frequency vectors across all MHCs is shown above each bar (ns: not significant, *p< 0.05, **p< 0.01, ***p< 0.001, p-values are adjusted for multiple testing using Benjamini-Hochberg correction). Amino acids are grouped by the properties of their side chains.

Supplementary Figure 20 Overview of the NetMHCpan-4.2 architecture. The input layer consists of a list of features related to the peptide and MHC molecule. The input features are connected to a single hidden layer, which in turn is connected to an output layer with two neurons predicting either binding affinity or eluted ligand likelihood. Orange boxes correspond to sequence inputs which are encoded using the BLOSUM50 matrix. Green boxes correspond to the novel features related to residue deletions. Note that the peptide context input is only used for the models trained with this feature.

Supplementary Figure 21 Cross-validated performance when training models using the final NetMHCpan-4.2 architecture with and without early stopping. In the ‘with early stopping’ method, each model was trained for up to 200 epochs, and the model with the lowest squared error In our cross-validation, the early stopping is done on the same test fold which is used to evaluate the models.

Supplementary Table 1 Overview of the included new EL samples and their alleles with low peptide counts in the old EL data.

Supplementary Table 2 Allelic frequencies from allelefrequencies.net. The frequencies are provided in the external file ‘table_s2.xlsx’.

Supplementary Table 3 Complete training data overview. The table is provided in the external file ‘table_s3.xlsx’.

Supplementary Table 4 Overview of datasets not included in the final NetMHCpan-4.2 training data. The table is provided in the external file ‘table_s4.xlsx’.

Supplementary Table 5 Overview of peptide-MHC structures obtained from the PDB database used for calculating positional and amino-acid specific interaction frequencies. The table is provided in the external file ‘table_s5.xlsx’.

## References

[1] Tynan FE Borg NA Miles JJ Beddoe T El-Hassen D Silins SL. High resolution structures of highly bulged viral epitopes bound to major histocompatibility complex class I: Implications for T-cell receptor engagement and T-cell immunodominance. J Biol Chem. (2005) 280:23900–9. doi: 10.1074/jbc.M503060200, PMID: 15849183

[2] Alvarez B Reynisson B Barra C Buus S Ternette N Connelley T. NNAlign_MA; MHC peptidome deconvolution for accurate MHC binding motif characterization and improved T-cell epitope predictions. Mol Cell Proteomics. (2019) 18:2459–77. doi: 10.1074/mcp.TIR119.001658, PMID: 31578220 PMC6885703

[3] Reynisson B Alvarez B Paul S Peters B Nielsen M. NetMHCpan-4.1 and NetMHCIIpan-4.0: improved predictions of MHC antigen presentation by concurrent motif deconvolution and integration of MS MHC eluted ligand data. Nucleic Acids Res. (2020) 48:W449–54. doi: 10.1093/nar/gkaa379, PMID: 32406916 PMC7319546

[4] Thrift WJ Perera J Cohen S Lounsbury NW Gurung HR Rose CM. Graph-pMHC: graph neural network approach to MHC class II peptide presentation and antibody immunogenicity. Brief Bioinform. (2024) 25(3):bbae123. doi: 10.1093/bib/bbae123, PMID: 38555476 PMC10981672

[5] Krishnaswamy S Givechian K Rocha J Yang E Liu C Greene K. ImmunoStruct: a multimodal neural network framework for immunogenicity prediction from peptide-MHC sequence, structure, and biochemical properties. Res Sq. (2025). doi: 10.21203/rs.3.rs-6606336/v1, PMID: 40470208 PMC12136731

[6] Borch A Carri I Reynisson B Alvarez HMG Munk KK Montemurro A. IMPROVE: a feature model to predict neoepitope immunogenicity through broad-scale validation of T-cell recognition. Front Immunol. (2024) 15:1360281. doi: 10.3389/fimmu.2024.1360281, PMID: 38633261 PMC11021644

[7] Wan Y-TR Koşaloğlu-Yalçın Z Peters B Nielsen M. A large-scale study of peptide features defining immunogenicity of cancer neo-epitopes. NAR Cancer. (2024) 6(1):zcae002. doi: 10.1093/narcan/zcae002, PMID: 38288446 PMC10823584

[8] Gfeller D Schmidt J Croce G Guillaume P Bobisse S Genolet R. Improved predictions of antigen presentation and TCR recognition with MixMHCpred2.2 and PRIME2.0 reveal potent SARS-CoV-2 CD8+ T-cell epitopes. Cell Syst. (2023) 14(1):72–83.e5. doi: 10.1016/j.cels.2022.12.002, PMID: 36603583 PMC9811684

[9] Calis JJA de Boer RJ Keşmir C. Degenerate T-cell recognition of peptides on MHC molecules creates large holes in the T-cell repertoire. PloS Comput Biol. (2012) 8(3):e1002412. doi: 10.1371/journal.pcbi.1002412, PMID: 22396638 PMC3291541

[10] Carri I Schwab E Trivino JC von Euw EM Nielsen M Mordoh J. VACCIMEL, an allogeneic melanoma vaccine, efficiently triggers T cell immune responses against neoantigens and alloantigens, as well as against tumor-associated antigens. Front Immunol. (2024) 15:1496204. doi: 10.3389/fimmu.2024.1496204, PMID: 39840067 PMC11747570

[11] Albert BA Yang Y Shao XM Singh D Smith KN Anagnostou V. Deep neural networks predict class I major histocompatibility complex epitope presentation and transfer learn neoepitope immunogenicity. Nat Mach Intell. (2023) 5:861–72. doi: 10.1038/s42256-023-00694-6, PMID: 37829001 PMC10569228

[12] Xu H Hu R Dong X Kuang L Zhang W Tu C. ImmuneApp for HLA-I epitope prediction and immunopeptidome analysis. Nat Commun. (2024) 15:8926. doi: 10.1038/s41467-024-53296-0, PMID: 39414796 PMC11484853

[13] O’Brien H Salm M Morton LT Szukszto M O’Farrell F Boulton C. A modular protein language modelling approach to immunogenicity prediction. PloS Comput Biol. (2024) 20(11):e1012511. doi: 10.1371/journal.pcbi.1012511, PMID: 39527593 PMC11581412

[14] Vita R Blazeska N Marrama D Shackelford D Zalman L Foos G. The immune epitope database (IEDB): 2024 update. Nucleic Acids Res. (2025) 53(D1):D436–43. doi: 10.1093/nar/gkae1092, PMID: 39558162 PMC11701597

[15] Kosaloglu-Yalcin Z Blazeska N Vita R Carter H Nielsen M Schoenberger S. The cancer epitope database and analysis resource (CEDAR). Nucleic Acids Res. (2023) 51(D1):D845–52. doi: 10.1093/nar/gkac902, PMID: 36250634 PMC9825495

[16] Gonzalez-Galarza FF McCabe A Dos Santos EJM Jones J Takeshita L Ortega-Rivera ND. Allele frequency net database (AFND) 2020 update: Gold-standard data classification, open access genotype data and new query tools. Nucleic Acids Res. (2020) 48(D1):D783–8. doi: 10.1093/nar/gkz1029, PMID: 31722398 PMC7145554

[17] Burley SK Berman HM Bhikadiya C Bi C Chen L Di Costanzo L. Protein Data Bank: The single global archive for 3D macromolecular structure data. Nucleic Acids Res. (2019) 47(D1):D520–8. doi: 10.1093/nar/gky949, PMID: 30357364 PMC6324056

[18] Egholm Bruun Jensen E Reynisson B Barra C Nielsen M. New light on the HLA-DR immunopeptidomic landscape. J Leukoc Biol. (2024) 115:913–25. doi: 10.1093/jleuko/qiae007, PMID: 38214568 PMC11057780

[19] Mirdita M Schütze K Moriwaki Y Heo L Ovchinnikov S Steinegger M. ColabFold: making protein folding accessible to all. Nat Methods. (2022) 19:679–82. doi: 10.1038/s41592-022-01488-1, PMID: 35637307 PMC9184281

[20] Schrödinger L DeLano W. The pyMOL Molecular Graphics System (2024). Available online at: http://www.pymol.org/pymol (Accessed July 23, 2025).

[21] Barra C Alvarez B Paul S Sette A Peters B Andreatta M. Footprints of antigen processing boost MHC class II natural ligand predictions. Genome Med. (2018) 10:84. doi: 10.1186/s13073-018-0594-6, PMID: 30446001 PMC6240193

[22] Reynisson B Barra C Kaabinejadian S Hildebrand WH Peters B Nielsen M. Improved prediction of MHC II antigen presentation through integration and motif deconvolution of mass spectrometry MHC eluted ligand data. J Proteome Res. (2020) 19:2304–15. doi: 10.1021/acs.jproteome.9b00874, PMID: 32308001

[23] Thrift WJ Lounsbury NW Broadwell Q Heidersbach A Freund E Abdolazimi Y. Towards designing improved cancer immunotherapy targets with a peptide-MHC-I presentation model, HLApollo. Nat Commun. (2024) 15:10752. doi: 10.1038/s41467-024-54887-7, PMID: 39737928 PMC11686168

[24] Nielsen M Andreatta M. NNAlign: a platform to construct and evaluate artificial neural network models of receptor-ligand interactions. Nucleic Acids Res. (2017) 45(W1):W344–9. doi: 10.1093/nar/gkx276, PMID: 28407117 PMC5570195

[25] Nielsen M Lundegaard C Blicher T Lamberth K Harndahl M Justesen S. NetMHCpan, a method for quantitative predictions of peptide binding to any HLA-A and -B locus protein of known sequence. PloS One. (2007) 2(8):e796. doi: 10.1371/journal.pone.0000796, PMID: 17726526 PMC1949492

[26] Bassani-Sternberg M Chong C Guillaume P Solleder M Pak HS Gannon PO. Deciphering HLA-I motifs across HLA peptidomes improves neo-antigen predictions and identifies allostery regulating HLA specificity. PloS Comput Biol. (2017) 13(8):e1005725. doi: 10.1371/journal.pcbi.1005725, PMID: 28832583 PMC5584980

[27] Nielsen M Lundegaard C Worning P Lauemoller SL Lamberth K Buus S. Reliable prediction of T-cell epitopes using neural networks with novel sequence representations. Protein Sci. (2003) 12:1007–17. doi: 10.1110/ps.0239403, PMID: 12717023 PMC2323871

[28] Dao T Klatt MG Korontsvit T Mun SS Guzman S Mattar M. Impact of tumor heterogeneity and microenvironment in identifying neoantigens in a patient with ovarian cancer. Cancer Immunol Immunother. (2021) 70:1189–202. doi: 10.1007/s00262-020-02764-9, PMID: 33123756 PMC8053669

[29] Forlani G Michaux J Pak HS Huber F Marie Joseph EL Ramia E. CIITA-transduced glioblastoma cells uncover a rich repertoire of clinically relevant tumor-associated HLA-II antigens. Mol Cell Proteomics. (2021) 20:100032. doi: 10.1074/MCP.RA120.002201, PMID: 33592498 PMC8724627

[30] Goncalves G Mullan KA Duscharla D Ayala R Croft NP Faridi P. IFNγ Modulates the immunopeptidome of triple negative breast cancer cells by enhancing and diversifying antigen processing and presentation. Front Immunol. (2021) 12:645770. doi: 10.3389/fimmu.2021.645770, PMID: 33968037 PMC8100505

[31] Marino F Semilietof A Michaux J Pak HS Coukos G Müller M. Biogenesis of HLA ligand presentation in immune cells upon activation reveals changes in peptide length preference. Front Immunol. (2020) 11:1981. doi: 10.3389/fimmu.2020.01981, PMID: 32983136 PMC7485268

[32] Murphy JP Yu Q Konda P Paulo JA Jedrychowski MP Kowalewski DJ. Multiplexed Relative Quantitation with Isobaric Tagging Mass Spectrometry Reveals Class I Major Histocompatibility Complex Ligand Dynamics in Response to Doxorubicin. Anal Chem. (2019) 91(8):5106–5115. doi: 10.1021/acs.analchem.8b05616.s002, PMID: 30779550 PMC7302430

[33] Ebrahimi-Nik H Michaux J Corwin WL Keller GLJ Shcheglova T Pak HS. Mass spectrometry–driven exploration reveals nuances of neoepitope-driven tumor rejection. JCI Insight. (2019) 4(14):e129152. doi: 10.1172/jci.insight.129152, PMID: 31219806 PMC6675551

[34] Sarkizova S Klaeger S Le PM Li LW Oliveira G Keshishian H. A large peptidome dataset improves HLA class I epitope prediction across most of the human population. Nat Biotechnol. (2020) 38:199–209. doi: 10.1038/s41587-019-0322-9, PMID: 31844290 PMC7008090

[35] Shinkawa T Tokita S Nakatsugawa M Kikuchi Y Kanaseki T Torigoe T. Characterization of CD8+ T-cell responses to non-anchor-type HLA class I neoantigens with single amino-acid substitutions. Oncoimmunology. (2021) 10(1):e187006. doi: 10.1080/2162402X.2020.1870062, PMID: 33537174 PMC7833734

[36] Shraibman B Barnea E Kadosh DM Haimovich Y Slobodin G Rosner I. Identification of tumor antigens among the HLA peptidomes of glioblastoma tumors and plasma. Mol Cell Proteomics. (2019) 18:1255–68. doi: 10.1074/mcp.RA119.001524, PMID: 31154438 PMC6553928

[37] Nielsen M Lundegaard C Lund O. Prediction of MHC class II binding affinity using SMM-align, a novel stabilization matrix alignment method. BMC Bioinf. (2007) 8:238. doi: 10.1186/1471-2105-8-238, PMID: 17608956 PMC1939856

[38] Robinson J Barker DJ Georgiou X Cooper MA Flicek P Marsh SGE. IPD-IMGT/HLA database. Nucleic Acids Res. (2020) 48(D1):D948–55. doi: 10.1093/nar/gkz950, PMID: 31667505 PMC7145640

[39] Lin T-Y Goyal P Girshick R He K Dollár P. (2017). Focal loss for dense object detection, in: 2017 IEEE International Conference on Computer Vision (ICCV), Venice, Italy: IEEE. pp. 2999–3007. doi: 10.1109/ICCV.2017.324

[40] Thomsen MCF Nielsen M. Seq2Logo: a method for construction and visualization of amino acid binding motifs and sequence profiles including sequence weighting, pseudo counts and two-sided representation of amino acid enrichment and depletion. Nucleic Acids Res. (2012) 40(W1):W281–7. doi: 10.1093/nar/gks469, PMID: 22638583 PMC3394285

