# Supplementary material for: NetMHCpan-4.2: improved prediction of CD8+ epitopes by use of transfer learning and structural features
Source: Front Immunol. 2025 Aug 7;16:1616113. doi: 10.3389/fimmu.2025.1616113 (PMC12367478; doi:10.3389/fimmu.2025.1616113)
Supplement: Supplementary Figure 1 — Motif deconvolution reveals high levels of ‘trash’ peptides in new datasets. Motif deconvolutions were performed for four newly analyzed single-allele datasets corresponding to HLA-A29:02, HLA-B54:01, HLA-B57:01, and HLA-C04:01, based on cross-validation predictions. The datasets were obtained from Sarkizova et al., 2020 (PMID: 31844290) and Mei et al., 2020 (PMID: 32357974). For each dataset, peptides with a %-rank >20 were annotated as ‘trash’, while those with a %-rank ≤20 were annotated as binders for the corresponding HLA molecule. Each row represents one dataset and shows, from left to right: the number of peptides assigned to the HLA and trash categories, peptide length distributions, the motif of predicted binders, and the motif of predicted trash peptides. [file DataSheet1.docx]

**Supplementary figures**


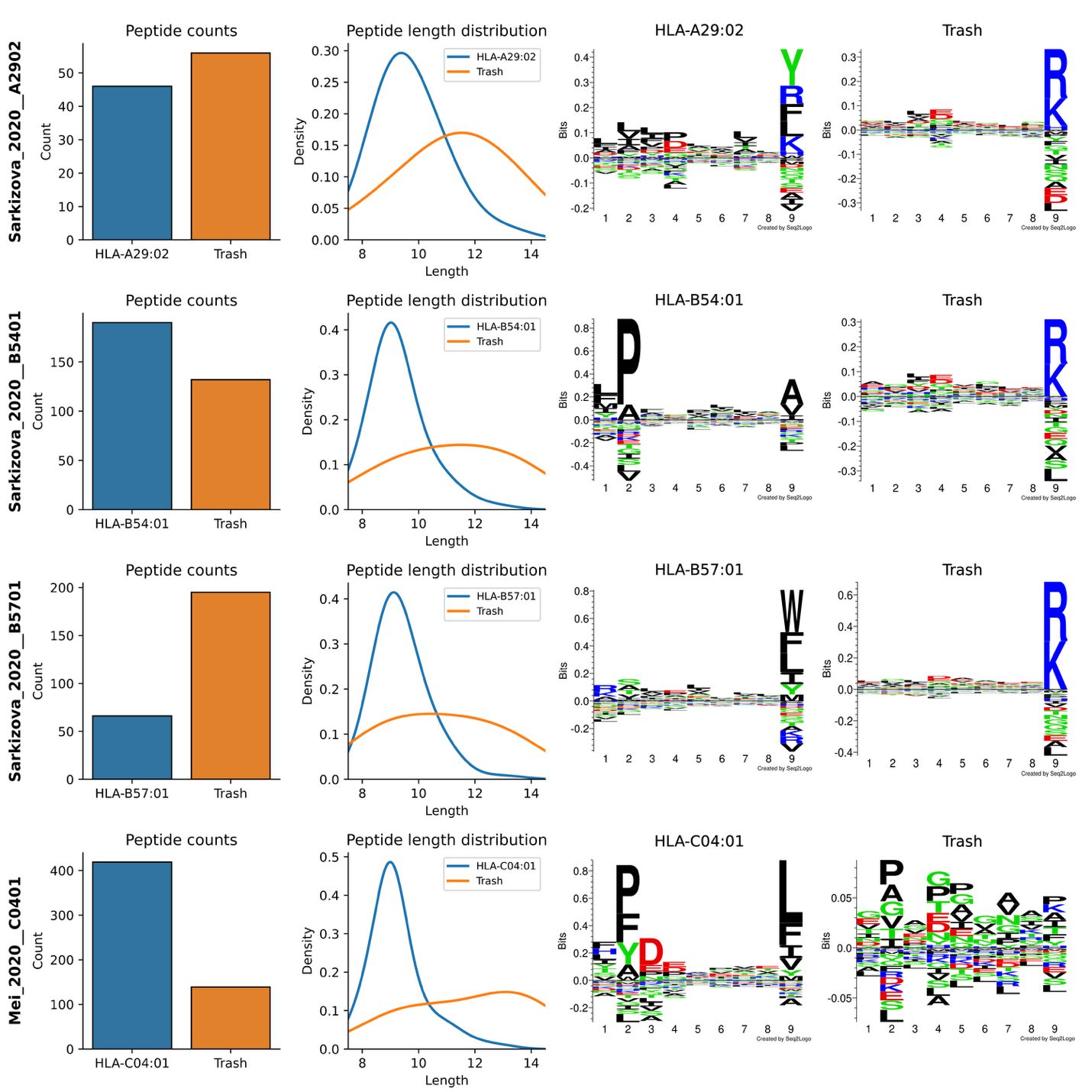


**Supplementary figure 1: Motif deconvolution reveals high levels of ‘trash’ peptides in new datasets.**

Motif deconvolutions were performed for four newly analyzed single-allele datasets corresponding to HLA-A29:02, HLA-B54:01, HLA-B57:01, and HLA-C04:01, based on cross-validation predictions. The datasets were obtained from Sarkizova et al. 2020 (PMID: 31844290) and Mei et al. 2020 (PMID: 32357974). For each dataset, peptides with a %-rank >20 were annotated as ‘trash’, while those with a %-rank ≤20 were annotated as binders for the corresponding HLA molecule. Each row represents one dataset and shows, from left to right: the number of peptides assigned to the HLA and trash categories, peptide length distributions, the motif of predicted binders, and the motif of predicted trash peptides.


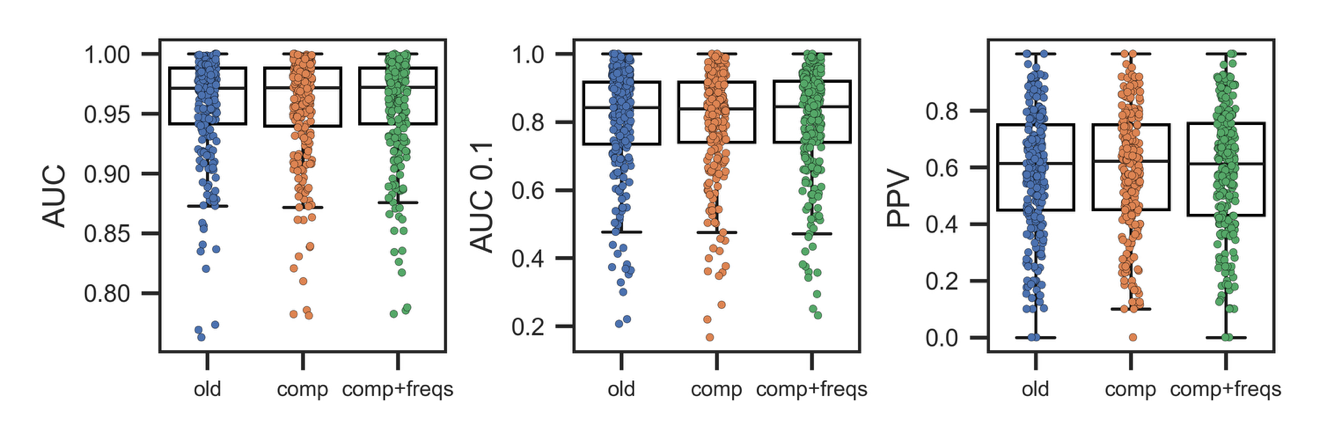


**Supplementary figure 2:** Cross-validated performance on peptides of length 8 for methods trained without new features (old), with average deletion composition (comp) and with average positional interaction frequency for deletion (comp+freqs). Each point is a dataset from the EL training data.
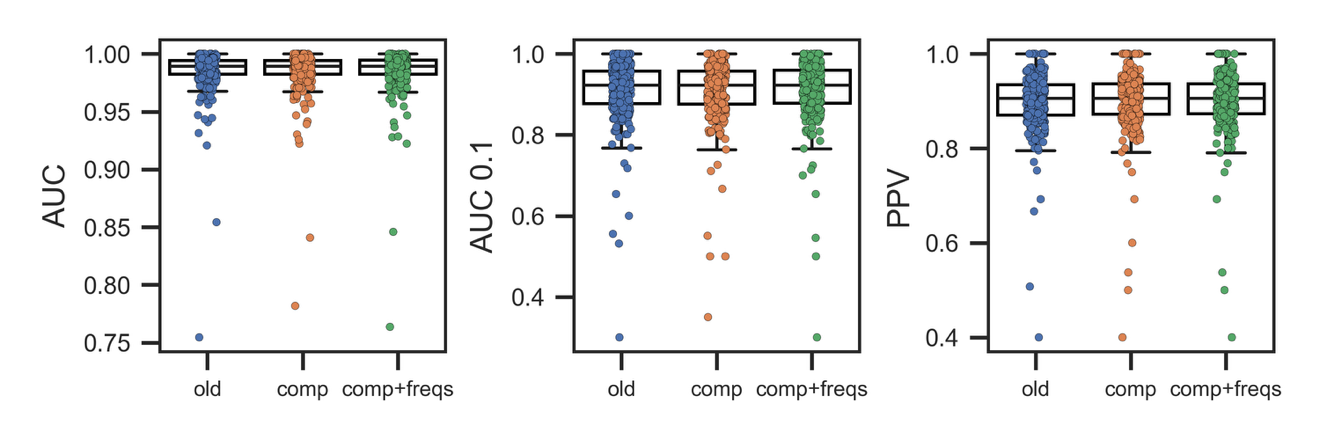


**Supplementary figure 3:** Cross-validated performance on peptides of length 9 for methods trained without new features (old), with average deletion composition (comp) and with average positional interaction frequency for deletion (comp+freqs). Each point is a dataset from the EL training data.
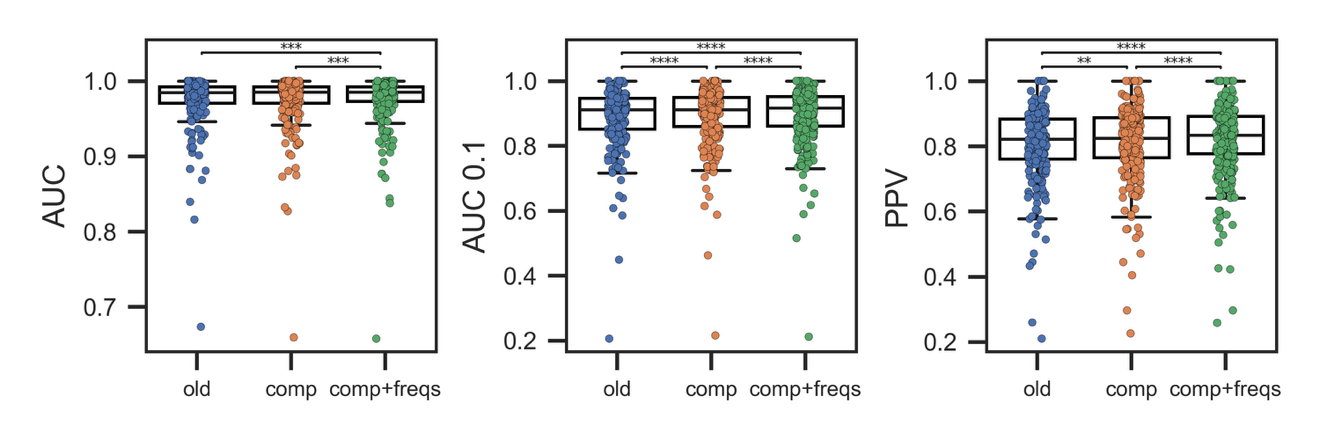


**Supplementary figure 4:** Cross-validated performance on peptides of length 10 for methods trained without new features (old), with average deletion composition (comp) and with average positional interaction frequency for deletion (comp+freqs). Each point is a dataset from the EL training data. Significant results from paired t-tests are shown (**: p < 0.01, ***: p < 0.001, ****: p < 0.0001).


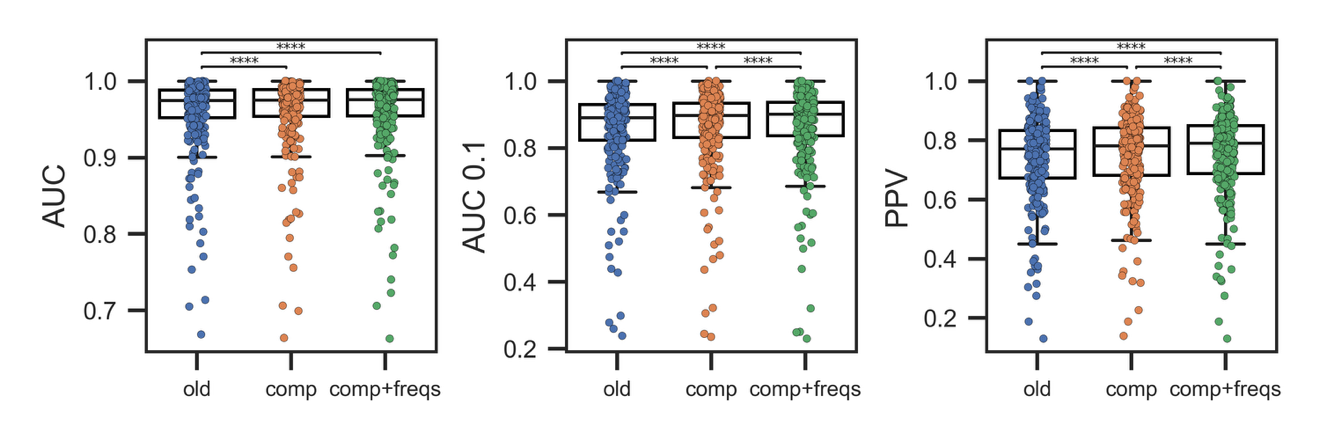


**Supplementary figure 5:** Cross-validated performance on peptides of length 11 for methods trained without new features (old), with average deletion composition (comp) and with average positional interaction frequency for deletion (comp+freqs). Each point is a dataset from the EL training data. Significant results from paired t-tests are shown (****: p < 0.0001).


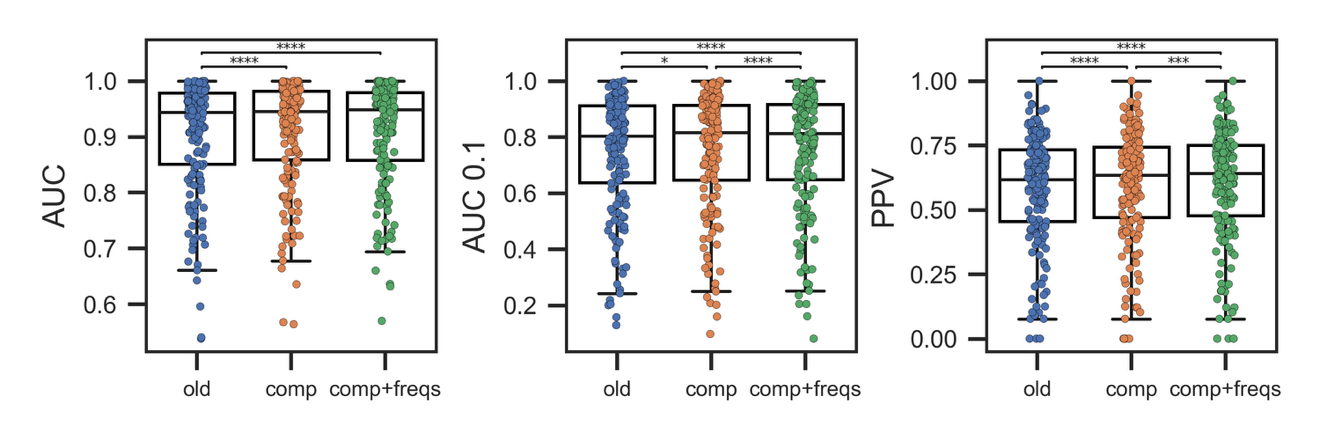


**Supplementary figure 6:** Cross-validated performance on peptides of length 12 for methods trained without new features (old), with average deletion composition (comp) and with average positional interaction frequency for deletion (comp+freqs). Each point is a dataset from the EL training data. Significant results from paired t-tests are shown (*: p < 0.05, ***: p < 0.001, ****: p < 0.0001).


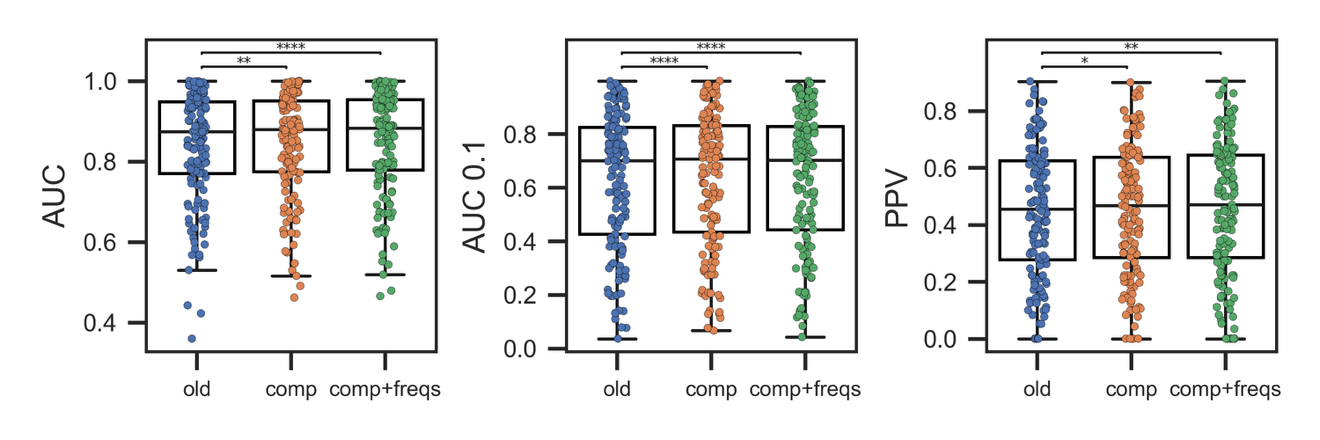


**Supplementary figure 7:** Cross-validated performance on peptides of length 13 for methods trained without new features (old), with average deletion composition (comp) and with average positional interaction frequency for deletion (comp+freqs). Each point is a dataset from the EL training data. Significant results from paired t-tests are shown (*: p < 0.05, **: p < 0.01, ****: p < 0.0001).


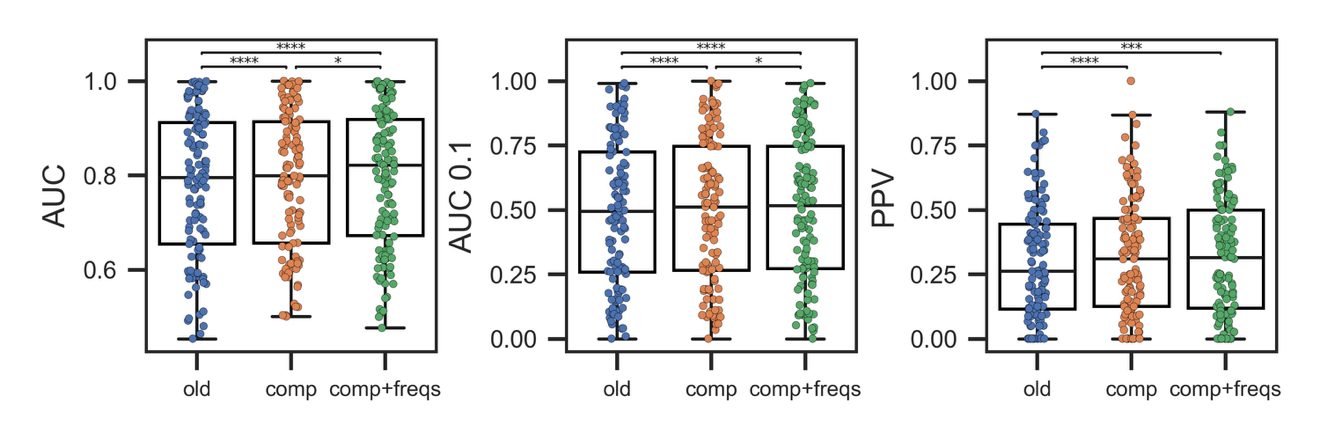


**Supplementary figure 8:** Cross-validated performance on peptides of length 14 for methods trained without new features (old), with average deletion composition (comp) and with average positional interaction frequency for deletion (comp+freqs). Each point is a dataset from the EL training data. Significant results from paired t-tests are shown (*: p < 0.05, ***: p < 0.001, ****: p < 0.0001).


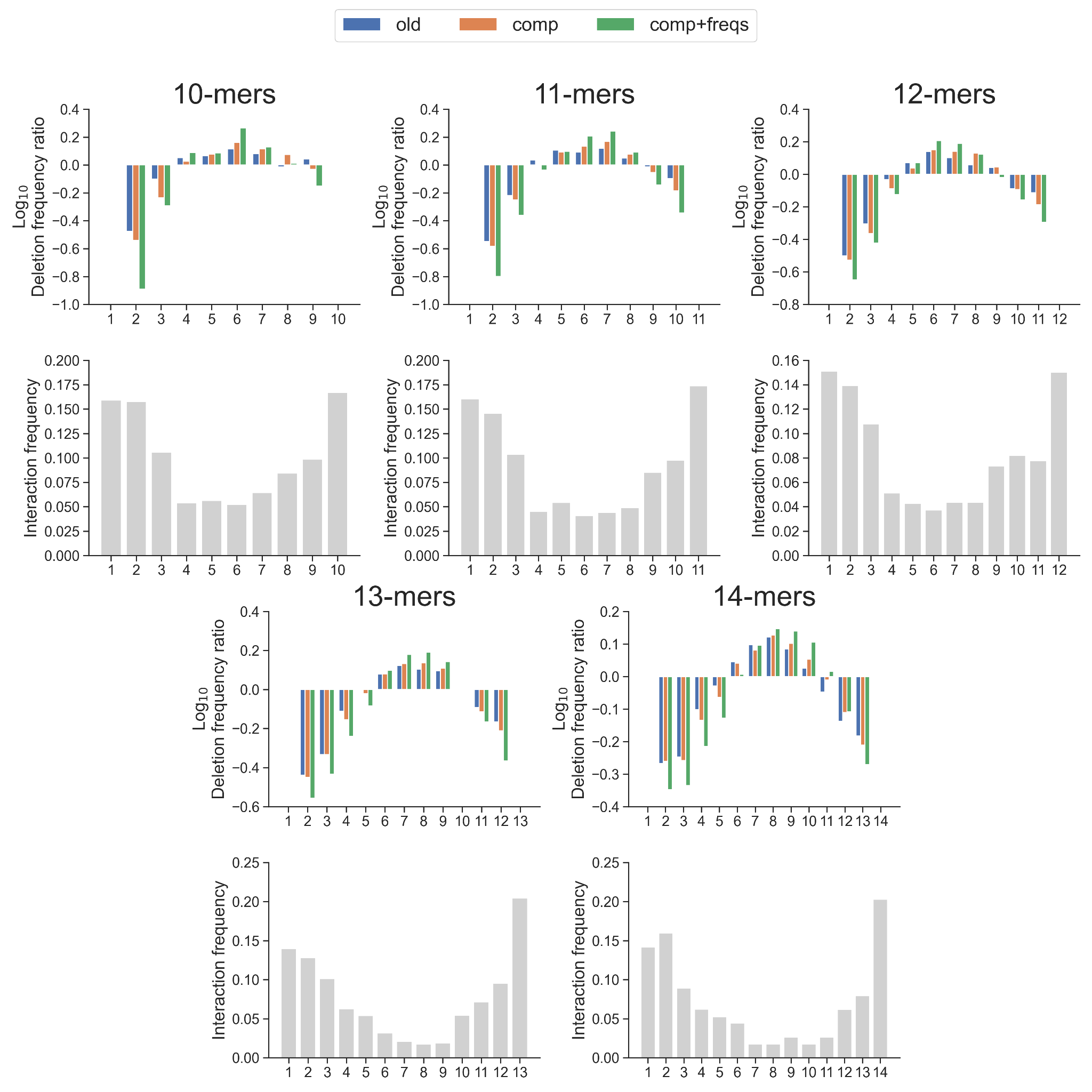


**Supplementary figure 9:** Log_10_ ratio of observed frequencies for deletion placements in 10-14-mers divided by frequencies obtained through random sampling of deletions in the same peptide set, for the methods without new features (old), with average deletion composition (comp) and with average deletion composition and positional interaction frequency for deletion (comp+freqs). Below each log_10_ frequency ratio plot, the positional interaction frequency vector for the given peptide length (which was used for the training of the comp+freqs method) is shown.


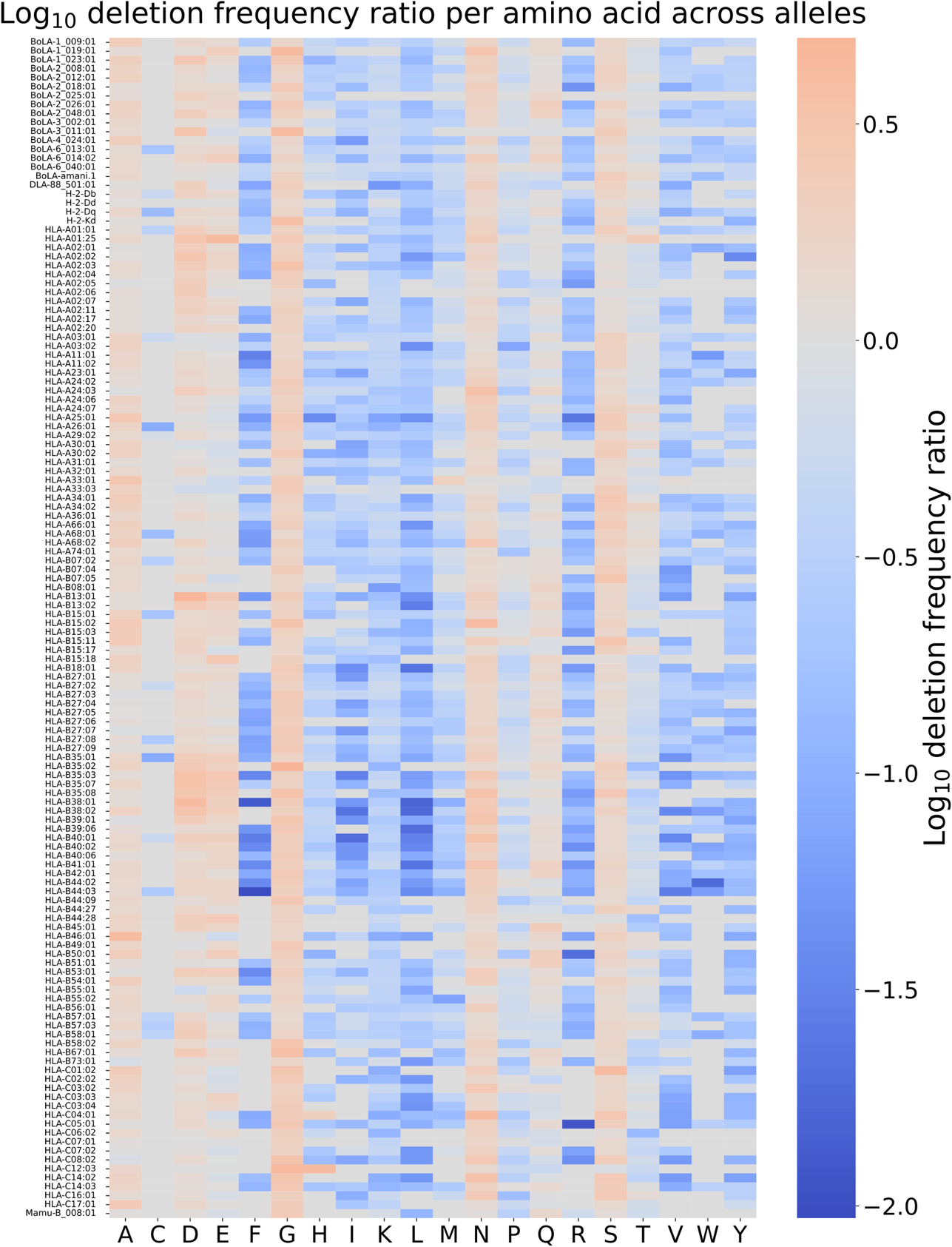


**Supplementary figure 10:** Log_10_ deletion frequency ratio per amino acid across alleles, calculated between the method with the new features and the method without the new features per amino acid. The ratios were calculated based on 10-14 mer peptides with improved %-rank in the method with the new features compared to the method without, and where the %-rank for the method with the new features was less than 2. Only alleles with at least 100 peptides were included.


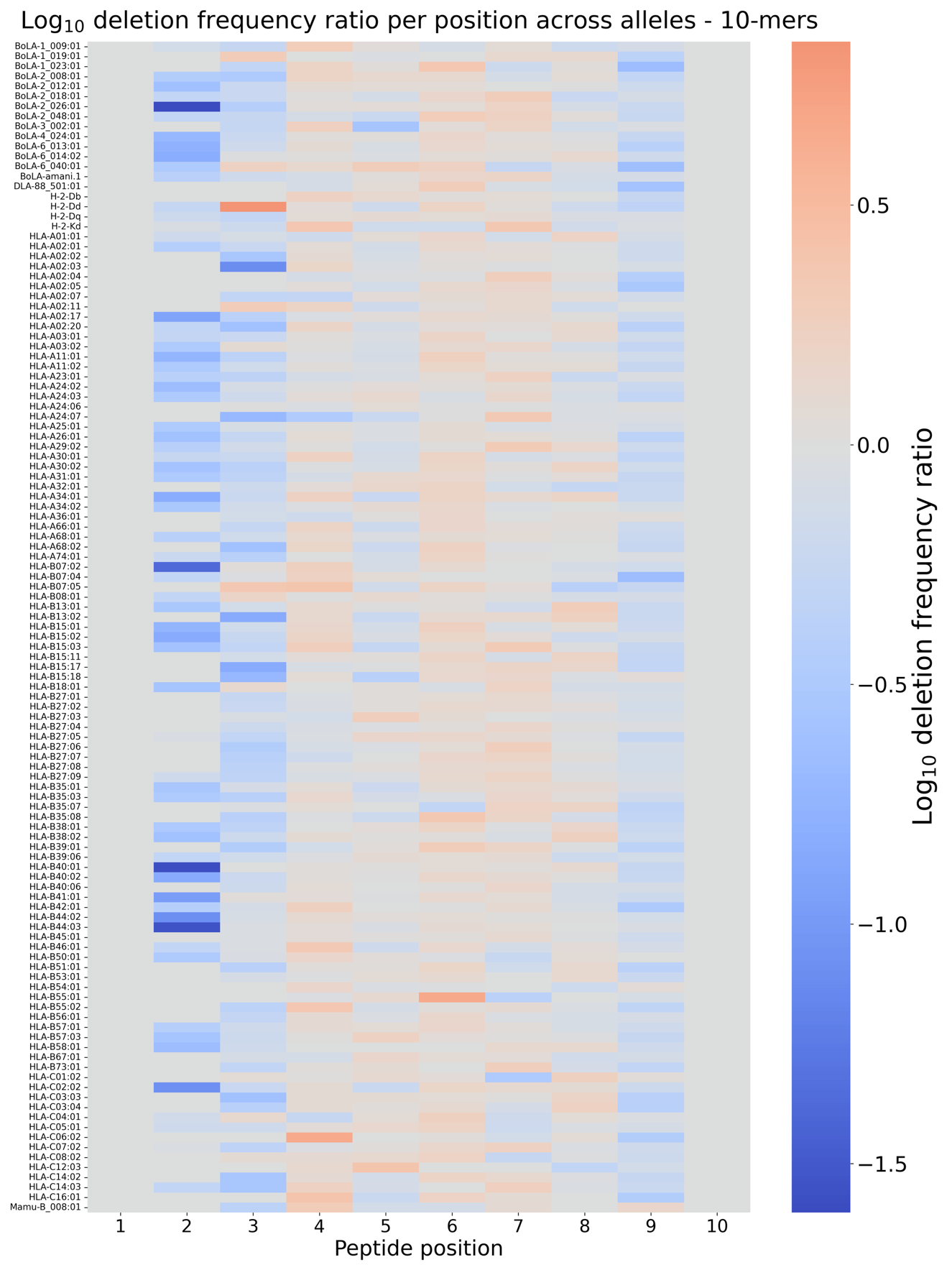


**Supplementary figure 11**: Log_10_ deletion frequency ratio per position in 10-mer peptides, calculated between the method with the new features and the method without the new features per amino acid. The ratios were calculated based on peptides with improved %-rank in the method with the new features compared to the method without, and where the %-rank for the method with the new features was less than 2. Only alleles with at least 100 peptides were included.


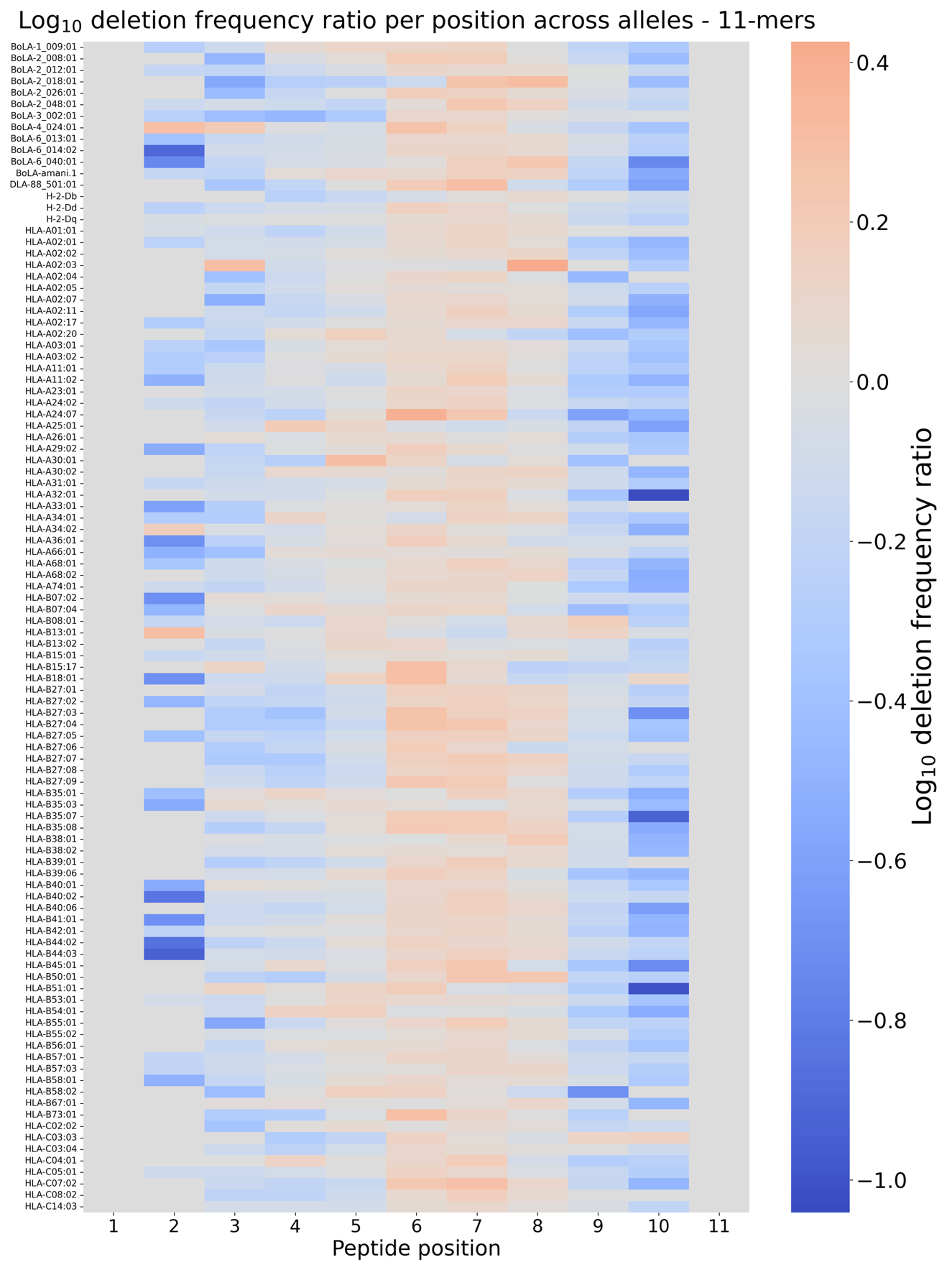


**Supplementary figure 12**: Log_10_ deletion frequency ratio per position in 11-mer peptides, calculated between the method with the new features and the method without the new features per amino acid. The ratios were calculated based on peptides with improved %-rank in the method with the new features compared to the method without, and where the %-rank for the method with the new features was less than 2. Only alleles with at least 100 peptides were included.


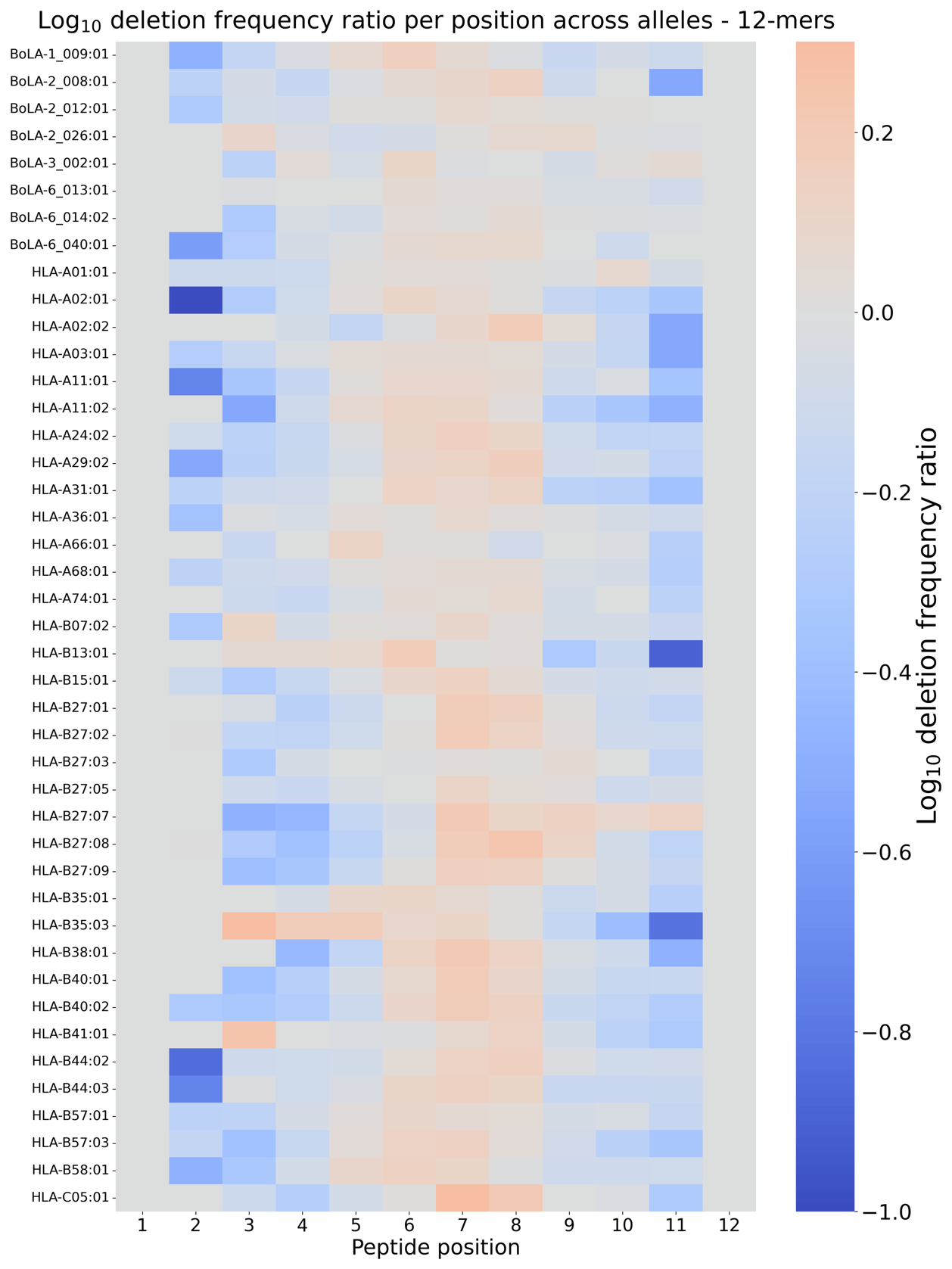


**Supplementary figure 13**: Log_10_ deletion frequency ratio per position in 12-mer peptides, calculated between the method with the new features and the method without the new features per amino acid. The ratios were calculated based on peptides with improved %-rank in the method with the new features compared to the method without, and where the %-rank for the method with the new features was less than 2. Only alleles with at least 100 peptides were included.


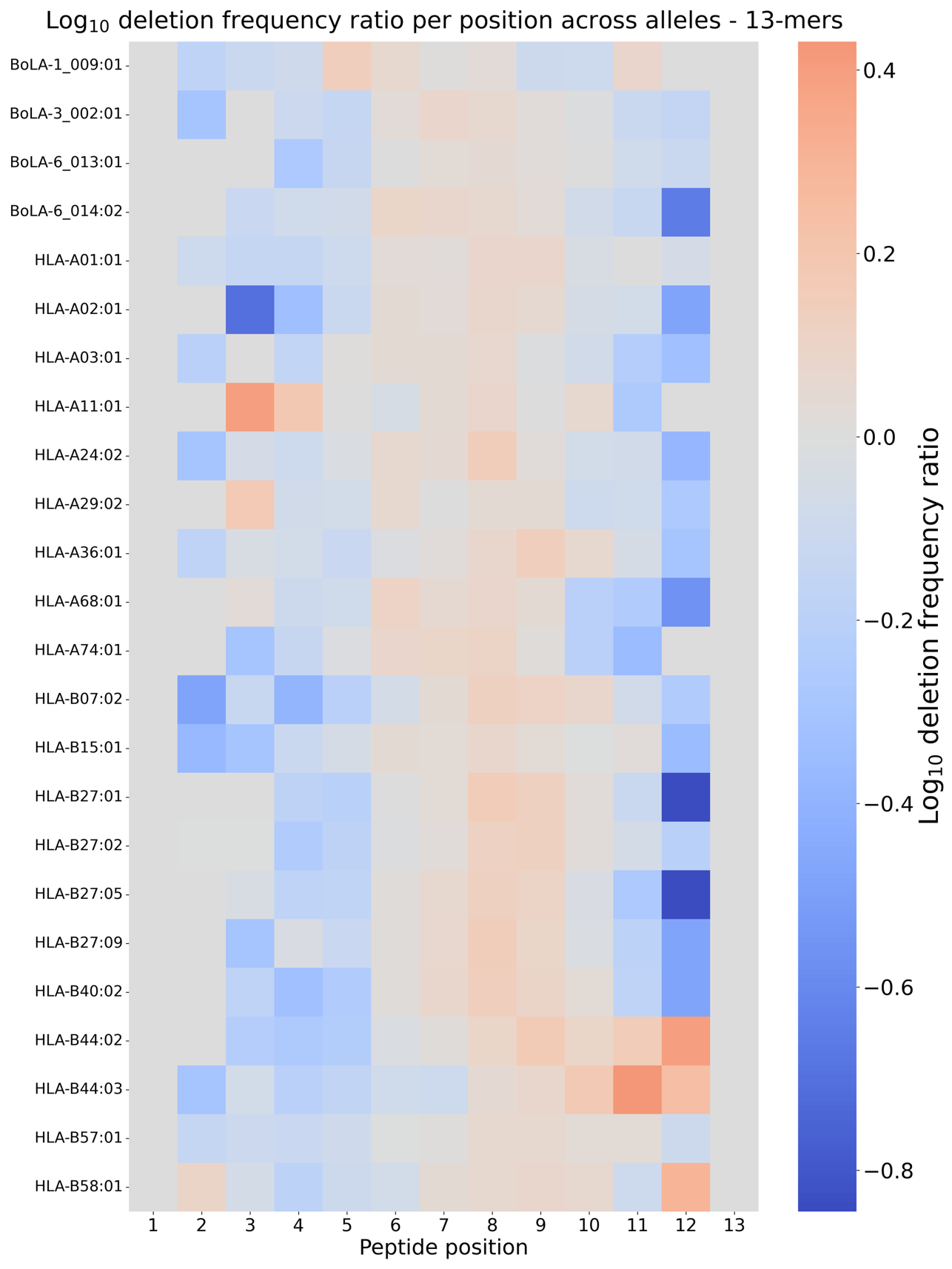


**Supplementary figure 14**: Log_10_ deletion frequency ratio per position in 13-mer peptides, calculated between the method with the new features and the method without the new features per amino acid. The ratios were calculated based on peptides with improved %-rank in the method with the new features compared to the method without, and where the %-rank for the method with the new features was less than 2. Only alleles with at least 100 peptides were included.


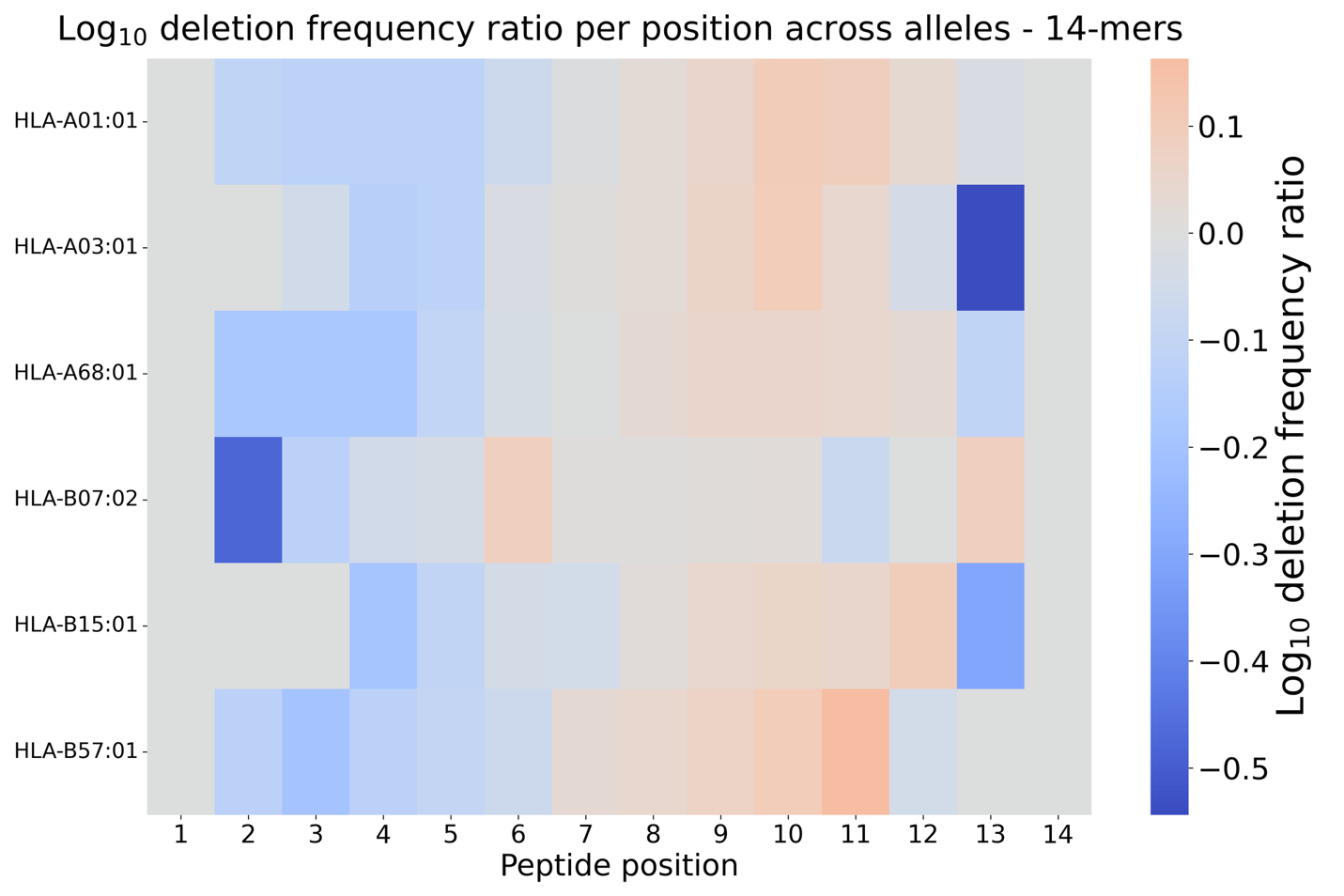


**Supplementary figure 15**: Log_10_ deletion frequency ratio per position in 14-mer peptides, calculated between the method with the new features and the method without the new features per amino acid. The ratios were calculated based on peptides with improved %-rank in the method with the new features compared to the method without, and where the %-rank for the method with the new features was less than 2. Only alleles with at least 100 peptides were included.


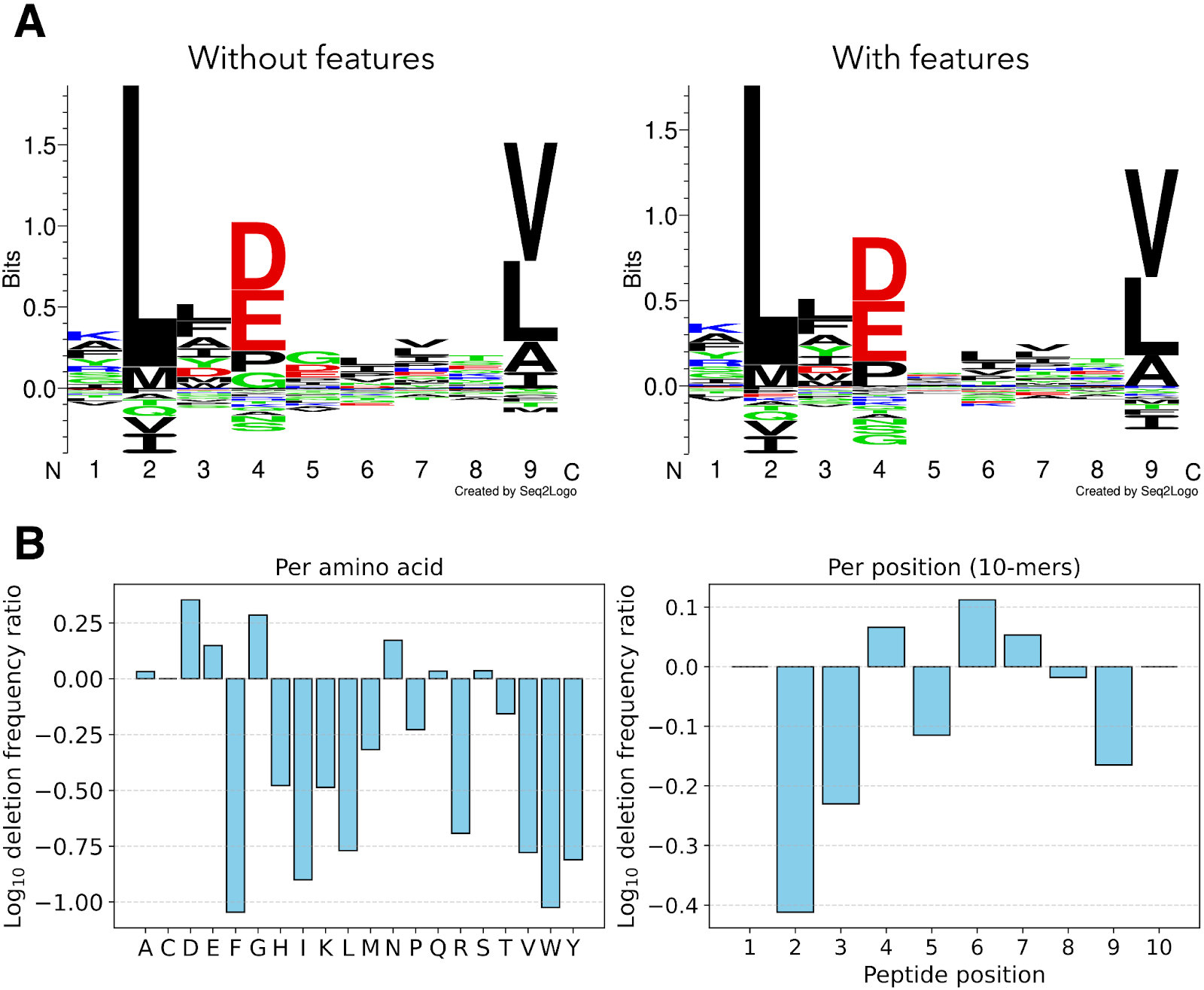

**Supplementary figure 16: Impact of new features on motif deconvolution for HLA-A02:01.** The plots are based on peptides with improved %-rank in the method with the new features compared to the method without, and where the %-rank for the method with the new features was less than 2**. A:** Sequence logos of predicted binding cores for the methods without and with the new features. **B:** Log_10_ deletion frequency ratio per amino acid (left) and per position in 10-mer peptides (right), calculated between the method with the new features and the method without the new features.


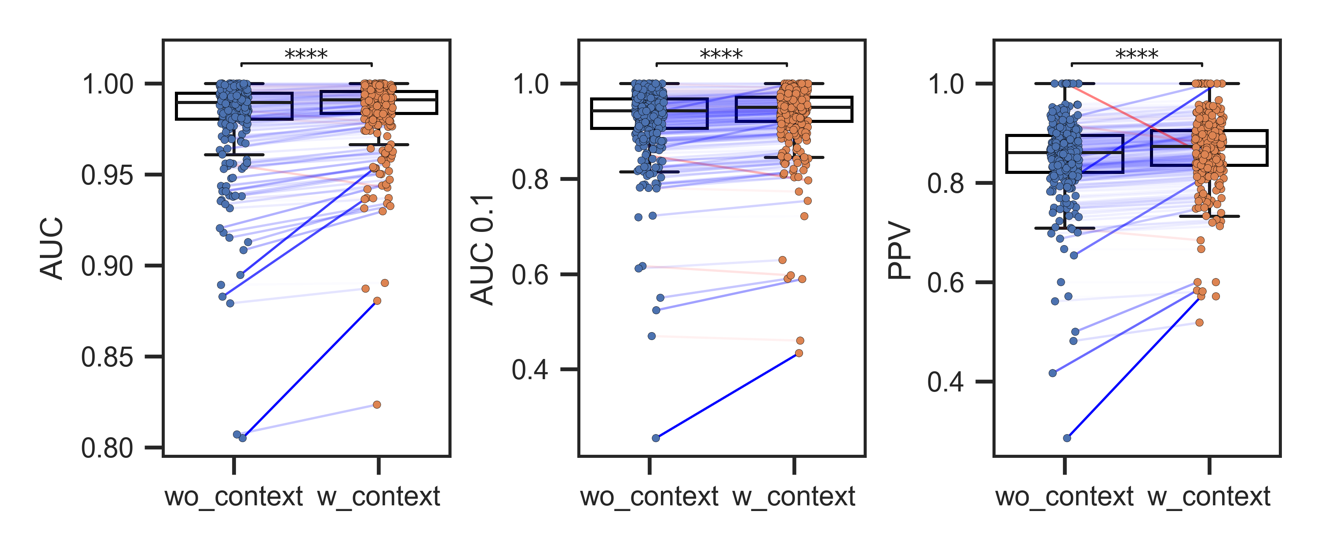


**Supplementary figure 17: Cross-validated performance of models trained without and with context encoding.** Each point is a dataset from the EL training data. Pairings corresponding to greater than or equal performance in the w_context method are colored blue and otherwise red (with the line weight indicating the magnitude of the difference). Significant results from paired t-tests are shown (****: p < 0.0001).

**
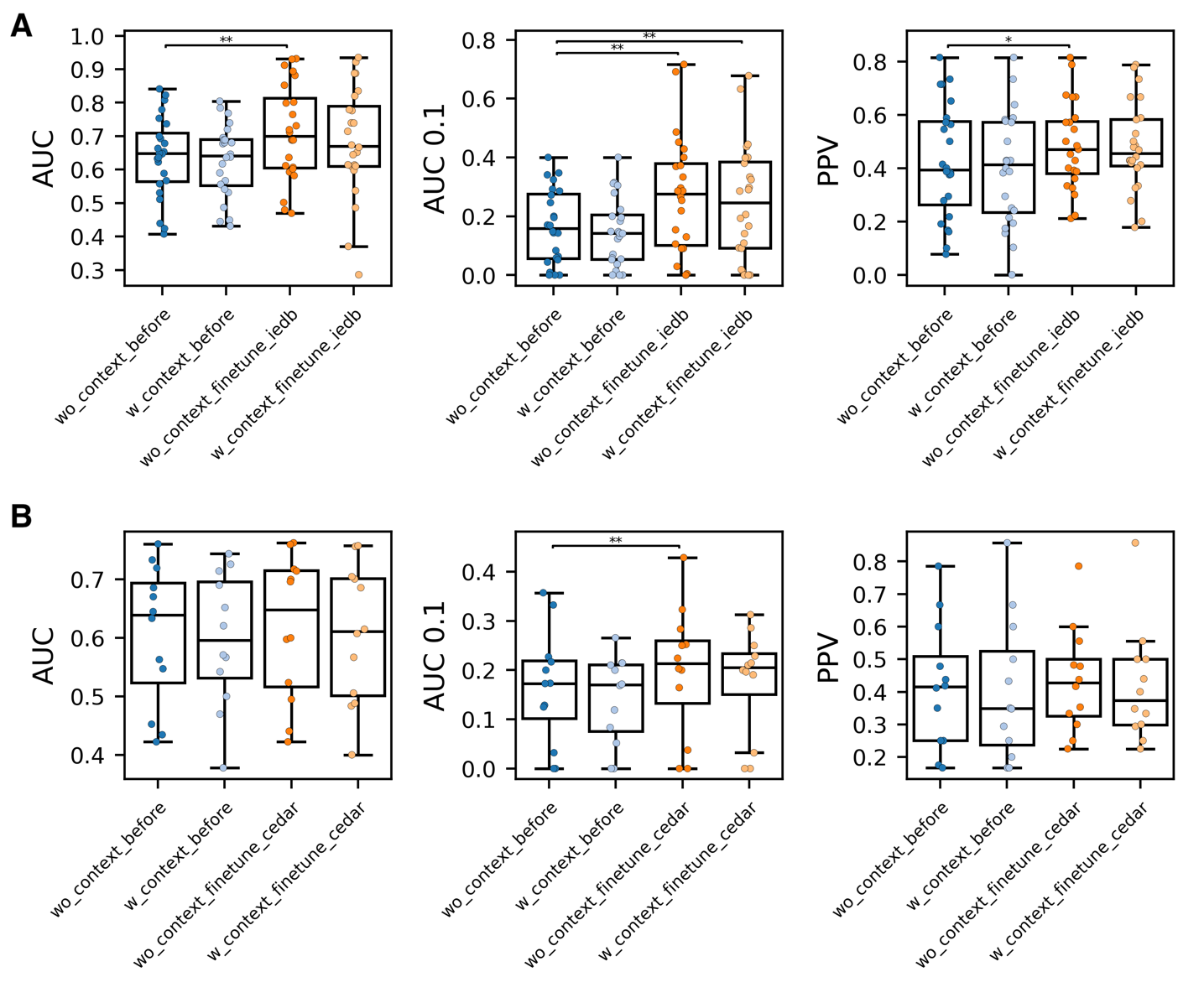
**

**Supplementary figure 18: Performance of models without and with context encoding on external epitope and neoepitope test sets. A:** Performance on external IEDB test set before (wo_context_before, w_context_before) and after (wo_context_finetune_iedb, w_context_finetune_iedb) fine-tuning on the IEDB training data. **B:** Performance on external CEDAR test set before (wo_context_before, w_context_before) and after (wo_context_finetune_cedar, w_context_finetune_cedar) fine-tuning on the CEDAR training data. In **A** and **B**, significant results from paired t-tests are shown (*: p < 0.05, **: p < 0.01).


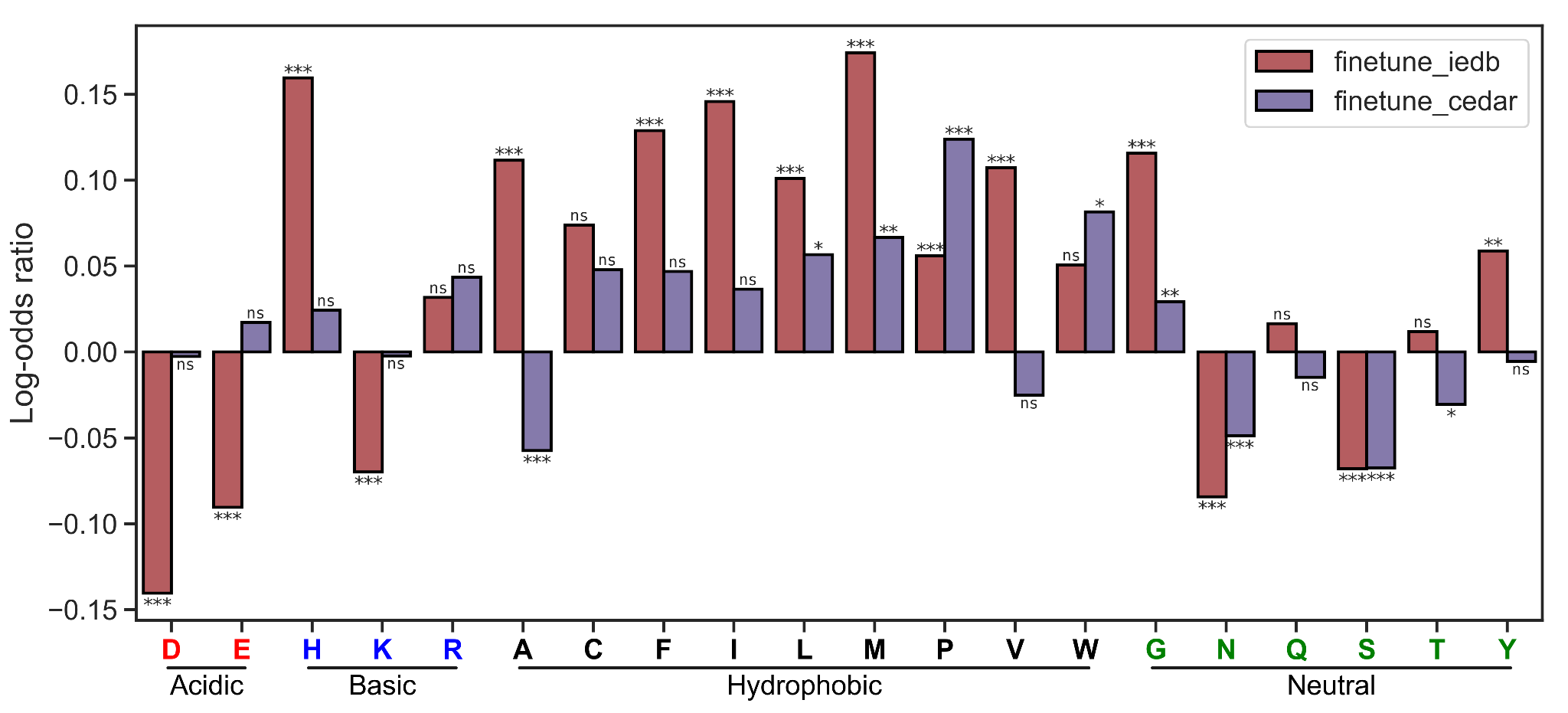


**Supplementary figure 19: Log-odds ratios of average amino acid frequencies in the deletions of top 1% of 75,000 random 10-12 mer peptides across all MHC molecules with unique pseudo-sequences in the IEDB and CEDAR training data.** Each bar is equal to the log_10_ of the average frequency in the refined method divided by the average frequency in the non-refined method. The result from a permutation test with 10,000 replications between the amino acid frequency vectors across all MHCs is shown above each bar (ns: not significant, *: p < 0.05, **: p < 0.01, ***: p < 0.001, p-values are adjusted for multiple testing using Benjamini-Hochberg correction). Amino acids are grouped by the properties of their side chains.


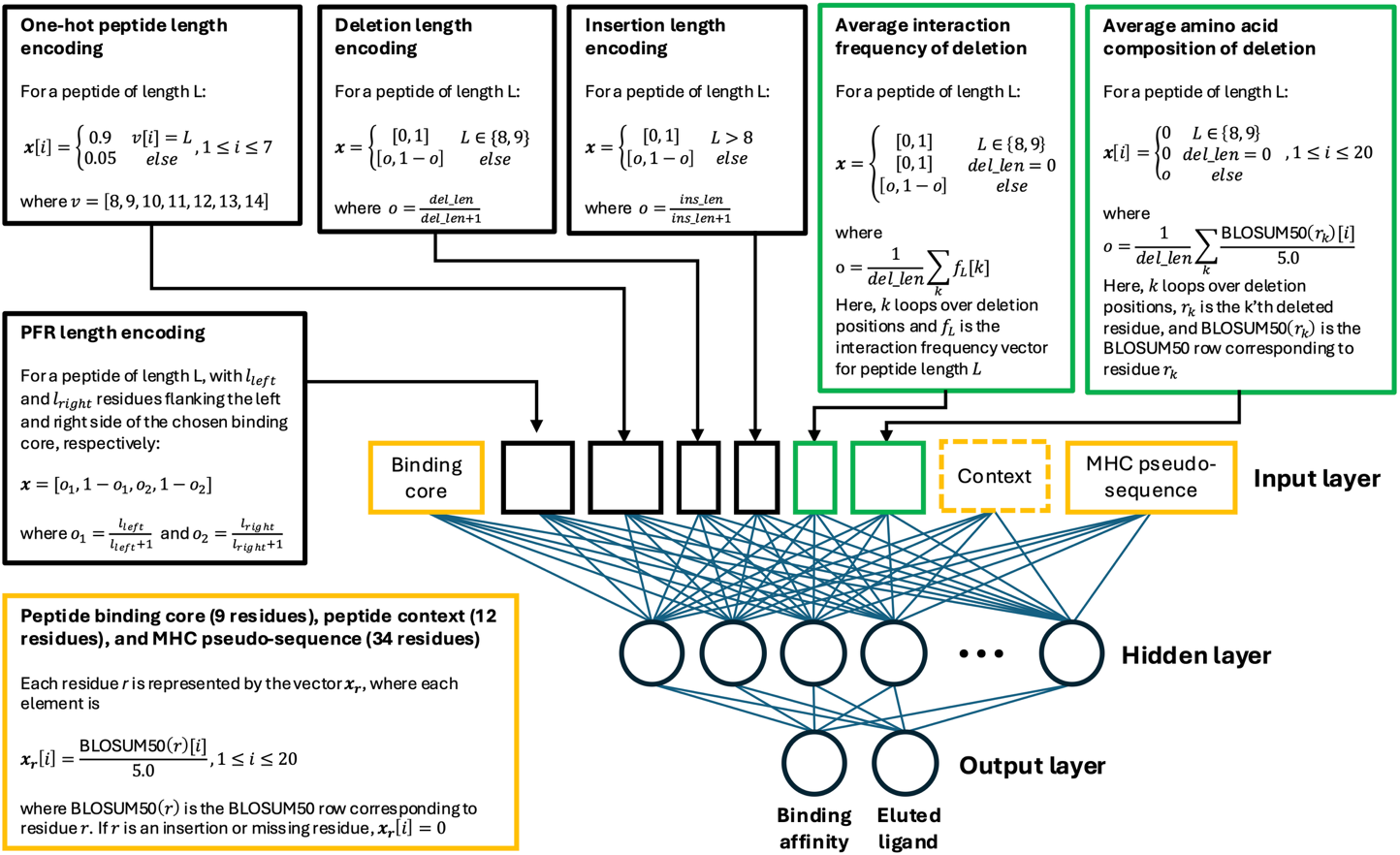

**Supplementary figure 20: Overview of the NetMHCpan-4.2 architecture.** The input layer consists of a list of features related to the peptide and MHC molecule. The input features are connected to a single hidden layer, which in turn is connected to an output layer with two neurons predicting either binding affinity or eluted ligand likelihood. Orange boxes correspond to sequence inputs which are encoded using the BLOSUM50 matrix. Green boxes correspond to the novel features related to residue deletions. Note that the peptide context input is only used for the models trained with this feature. Abbreviations: del_len: deletion length, ins_len: insertion length.


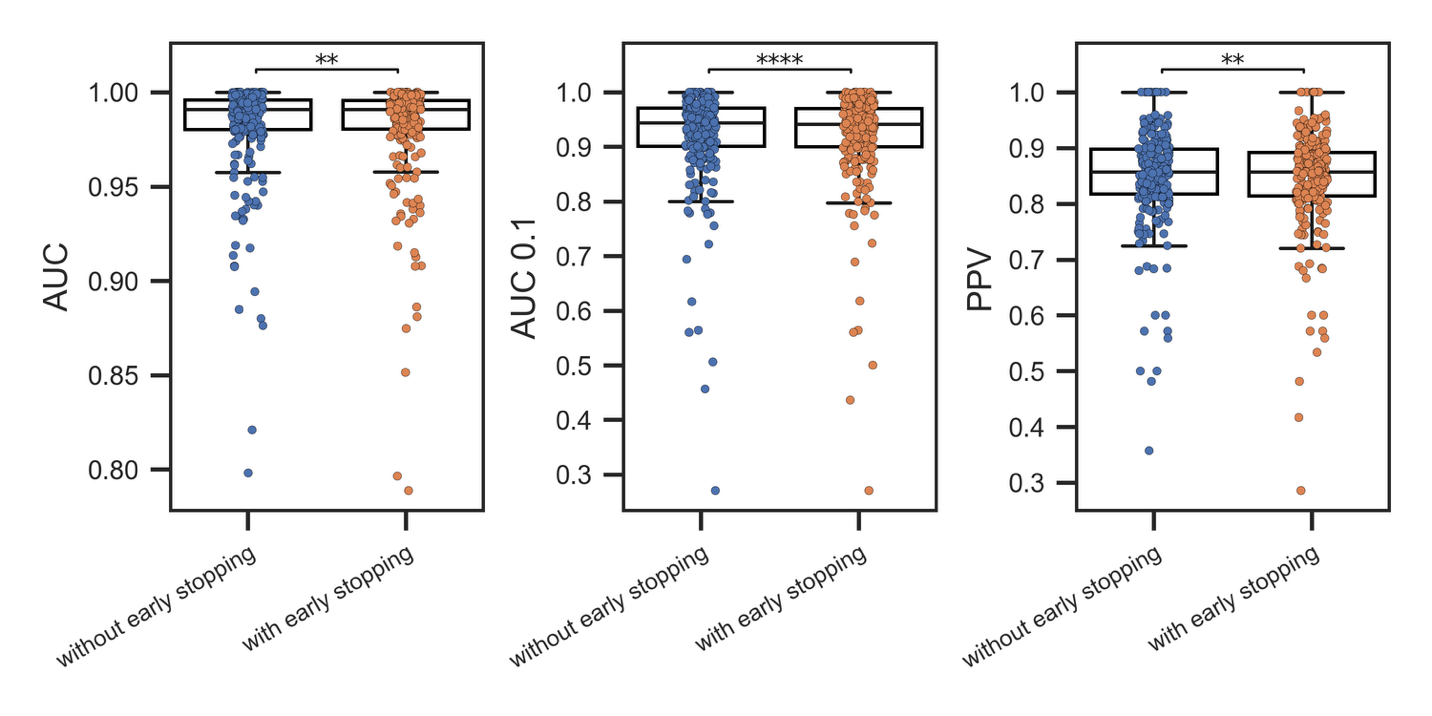


**Supplementary figure 21:** Cross-validated performance when training models using the final NetMHCpan-4.2 architecture with and without early stopping. In the ‘with early stopping’ method, each model was trained for up to 200 epochs, and the model with the lowest squared error In our cross-validation, the early stopping is done on the same test fold which is used to evaluate the models. Significant results from paired t-tests are shown (**: p < 0.01, ****: p < 0.0001).

**Supplementary tables**

**Supplementary table 1: Overview of the included new EL samples and their alleles with low peptide counts in the old EL data.**

| **Sample name** | **Alleles with low counts** | **Peptide counts in old EL data** |
| --- | --- | --- |
| Dao_2020__ovarian_cancer | HLA-B07:05 | 0 |
| Forlani_2021__HROG17 | HLA-A66:01 | 43 |
| Forlani_2021__HROG17_CIITA | HLA-A66:01 | 43 |
| Goncalves_2021__MDA_MB_231 | HLA-A02:17 | 0 |
| Marino_2020__D3 | HLA-C07:21 | 0 |
| Murphy_2019__MDAMB468 | HLA-B53:01 | 0 |
| Nik_2019__MethA | H-2-Dd, H-2-Ld | 0, 2 |
| Sarkizova_2020__A0202 | HLA-A02:02 | 0 |
| Sarkizova_2020__A0211 | HLA-A02:11 | 0 |
| Sarkizova_2020__A1102 | HLA-A11:02 | 0 |
| Sarkizova_2020__A2407 | HLA-A24:07 | 0 |
| Sarkizova_2020__A3401 | HLA-A34:01 | 0 |
| Sarkizova_2020__A3402 | HLA-A34:02 | 0 |
| Sarkizova_2020__A3601 | HLA-A36:01 | 0 |
| Sarkizova_2020__A6601 | HLA-A66:01 | 43 |
| Sarkizova_2020__A7401 | HLA-A74:01 | 0 |
| Sarkizova_2020__B0704 | HLA-B07:04 | 0 |
| Sarkizova_2020__B1301 | HLA-B13:01 | 63 |
| Sarkizova_2020__B1517 | HLA-B15:17 | 13 |
| Sarkizova_2020__B3507 | HLA-B35:07 | 0 |
| Sarkizova_2020__B3802 | HLA-B38:02 | 0 |
| Sarkizova_2020__B4006 | HLA-B40:06 | 0 |
| Sarkizova_2020__B4201 | HLA-B42:01 | 10 |
| Sarkizova_2020__B5201 | HLA-B52:01 | 98 |
| Sarkizova_2020__B5301 | HLA-B53:01 | 0 |
| Sarkizova_2020__B5502 | HLA-B55:02 | 5 |
| Sarkizova_2020__B5802 | HLA-B58:02 | 7 |
| Sarkizova_2020__C0302 | HLA-C03:02 | 0 |
| Sarkizova_2020__C0403 | HLA-C04:03 | 0 |
| Sarkizova_2020__C1403 | HLA-C14:03 | 0 |
| Sarkizova_2020__G0101 | HLA-G01:01 | 0 |
| Sarkizova_2020__G0103 | HLA-G01:03 | 0 |
| Sarkizova_2020__G0104 | HLA-G01:04 | 0 |
| Sarkizova_2020__MEL15 | HLA-A02:02 | 0 |
| Shinkawa_2021__HCT15 | HLA-C07:06 | 0 |
| Shraibman_2019__30_002 | HLA-B53:01 | 0 |
| Shraibman_2019__638_13 | HLA-A24:03, HLA-B15:17 | 0, 13 |
| Shraibman_2019__BCN_019 | HLA-B15:24 | 0 |
| Shraibman_2019__CPH_03 | HLA-A34:02, HLA-C18:01 | 0, 0 |
| Shraibman_2019__CPH_06 | HLA-A01:25 | 0 |
| Shraibman_2019__Leiden_002 | HLA-B07:05 | 0 |

**Supplementary table 2: Allelic frequencies from allelefrequencies.net.** The frequencies are provided in the external file ‘table_s2.xlsx’.

**Supplementary table 3: Complete training data overview.** The table is provided in the external file ‘table_s3.xlsx’.

**Supplementary table 4: Overview of datasets not included in the final NetMHCpan-4.2 training data.** The table is provided in the external file ‘table_s4.xlsx’.

**Supplementary table 5: Overview of peptide-MHC structures obtained from the PDB database used for calculating positional and amino-acid specific interaction frequencies.** The table is provided in the external file ‘table_s5.xlsx’.
